# Strain-specific strategies underlie convergent phosphate solubilization in *Bacillus*

**DOI:** 10.1093/ismeco/ycaf208

**Published:** 2025-11-14

**Authors:** Stefanie Katharina Thaqi, Roberto Siani, Akane Chiba, Manuela Peine, Christel Baum, Michael Witting, Susanne Walch, Peter Leinweber, Michael Schloter, Stefanie Schulz

**Affiliations:** Technische Universität München, TUM School of Life Sciences, Chair of Crop Physiology, 85354 Freising, Bavaria, Germany; Technische Universität München, TUM School of Life Sciences, Chair of Environmental Microbiology, 85354 Freising, Bavaria, Germany; Technische Universität München, TUM School of Life Sciences, Chair of Environmental Microbiology, 85354 Freising, Bavaria, Germany; Helmholtz Zentrum München, Research Unit Comparative Microbiome Analysis, 85764 Neuherberg, Bavaria, Germany; Technische Universität München, TUM School of Life Sciences, Chair of Environmental Microbiology, 85354 Freising, Bavaria, Germany; University of Rostock, Chair of Soil Science, 18059 Rostock, Mecklenburg-Western Pomerania, Germany; University of Rostock, Chair of Soil Science, 18059 Rostock, Mecklenburg-Western Pomerania, Germany; Helmholtz Zentrum München, Metabolomics and Proteomics Core, 85764 Neuherberg, Bavaria, Germany; Technische Universität München, TUM School of Life Sciences, Chair of Analytical Food Chemistry, 85354 Freising, Bavaria, Germany; Helmholtz Zentrum München, Research Unit Comparative Microbiome Analysis, 85764 Neuherberg, Bavaria, Germany; University of Rostock, Chair of Soil Science, 18059 Rostock, Mecklenburg-Western Pomerania, Germany; Technische Universität München, TUM School of Life Sciences, Chair of Environmental Microbiology, 85354 Freising, Bavaria, Germany; Helmholtz Zentrum München, Research Unit Comparative Microbiome Analysis, 85764 Neuherberg, Bavaria, Germany; Helmholtz Zentrum München, Research Unit Comparative Microbiome Analysis, 85764 Neuherberg, Bavaria, Germany

**Keywords:** phosphorus, bone char, bioinoculum, phosphorus-solubilizing bacteria, sustainable agriculture, metabolomics, transcriptomics, *Bacillus* spp

## Abstract

The stability of ecosystem functions under changing environmental conditions is often attributed to convergent functioning, where different mechanisms lead to similar outcomes. In soil systems, microbial activity is a major driver of nutrient cycling, yet it remains unclear whether the presence of the same genes across taxa reliably translates into redundant outcomes. We addressed this in microbial phosphate solubilization, critical when applying alternative phosphorus (P) fertilizers such as BC^plus^, a biochar-based fertilizer from pyrolyzed animal bones coated with sulfur. Using multi-omics analyses, we compared two soil isolates—*Bacillus licheniformis* COM1 and *Psychrobacillus psychrodurans* INOP01—alongside the reference strain *Bacillus velezensis* DSM 23117. *P. psychrodurans* was excluded due to poor growth under P limitation. Despite similar growth and P mobilization, *B. licheniformis* and *B. velezensis* relied on distinct strategies, indicating that mechanistically diverse regulatory programs can yield convergent phosphate-solubilizing outcomes. Transcriptional changes extended beyond P metabolism, with both strains inducing nitrate reduction and adjusting sulfur metabolism, underscoring tight coupling of P, nitrogen, and sulfur cycling. *B. velezensis* responded rapidly by inducing Pho genes, organic acid production, nitrate respiration, and plant growth–promoting traits including indole-3-acetic acid biosynthesis. *B. licheniformis* instead showed a slower adaptation marked by malate-driven acidification, dissimilatory nitrate reduction to ammonium, and late riboflavin activation. While both strains solubilized phosphate, their mechanisms differed, illustrating that convergence at the functional outcome does not imply similarity in regulation or metabolism. These results highlight the need to account for strain-specific pathways when developing microbial inoculants to optimize nutrient turnover in low-input systems.

## Introduction

Phosphorus (P) is vital for plant growth, but only 0.02%–0.2% of total soil P is readily accessible to plants [1]. This limited availability is primarily due to the precipitation of mineral P and sorption onto charged soil surfaces.

Although mineral P fertilizers offer an immediate source of plant-available P, their overuse contributes to eutrophication of water resources and depletes finite P reserves [2]. With global agricultural demand for P expected to increase by up to 86% by 2050, and additional losses from soil erosion [3, 4], there is an increasing interest in sustainable alternatives in P management.

These alternatives—such as P-recycling fertilizers like struvite or biochar from bones or sewage sludge, and organic amendments—typically contain poorly soluble or organic P forms, so their effectiveness largely depends on microbial activity to render P plant-available [5].

Among these microorganisms, P solubilizing bacteria (PSB) are particularly significant, as they contribute to increasing P availability and have been shown to improve crop yields [6–8]. The most common strategy involves acidifying the surrounding environment, typically through organic acid production or proton release. However, P solubilization is a multifaceted process. Additional mechanisms include proton secretion via cation exchange, H^+^-translocating ATPases, and nitrogen (N) assimilation-linked acidification [9, 10]. PSB are taxonomically diverse, including genera such as *Pseudomonas* and *Bacillus* [11, 12]. Phosphate solubilization represents one of their major plant growth-promoting (PGP) mechanisms, as it enhances nutrient availability to plants.

Another key microbial mechanism to increase phosphorus availability under neutral to alkaline conditions is sulfur (S) oxidation. Here, microorganisms convert reduced and elemental S into sulfuric acid, thereby lowering the pH. This principle forms the basis of the so-called *in situ* “digestion” approach [13], in which elemental S is co-applied with rock phosphate fertilizers. The resulting acidification facilitates P release and can be further enhanced by inoculation with S-oxidizing bacteria [14]. A promising example of a fertilizer that makes use of this principle is S-enriched bone char (BC^plus^). Produced from pyrolyzed animal bones, BC^plus^ contains ~8% P, 27% S, 7% Ca, and 9% Mg, with enhanced solubility due to surface enrichment of a multitude of reduced S compounds [15]. Its effectiveness depends strongly on microbial action, particularly under neutral to alkaline soil conditions where apatite solubility is low.

Despite its S coating, BC^plus^ remains a low-solubility, slow-release phosphorus fertilizer, with significantly lower solubility than Triplesuperphosphate, struvite, or ash-based fertilizers [16]. This gradual release may support long-term nutrient availability but requires stimulation of a functionally diverse soil microbiome to be effective. Field data suggest that BC^plus^ can provide yield benefits compared to unfertilized controls under P-deficient conditions, but only after several years of repeated application [17]. The delayed effectiveness of BC^plus^ suggests potential for improved solubility via microbial mediation.

Interestingly, an increased abundance of *gcd*-harboring bacteria following BC^plus^ application has been reported [18]. The *gcd* gene encodes a glucose dehydrogenase, an enzyme involved in the production of gluconic acid, a key metabolite for inorganic phosphate solubilization. This suggests that BC^plus^ may selectively enrich PSB. We hypothesized that strains with different niche histories would deploy distinct transcriptional and metabolic programs in response to BC^plus^ yet still achieve a convergent phosphate-solubilizing outcome. We further expected these programs to reflect regulatory trade-offs that are relevant for inoculant design and nutrient mobilization in low-input systems.

To test this hypothesis, we investigated whether selected PSB strains can improve the efficiency of BC^plus^-mediated P solubilization and aimed to analyze the underlying molecular mechanisms. We isolated PSB strains from soils enriched with compost-derived organic matter and from long-term P-unfertilized soils [19, 20], and compared them with a commercial bioinoculant strain, *Bacillus velezensis* DSM 23117. Given their distinct ecological origins, we expected the strains selected for our analysis to exhibit different physiological and molecular responses to BC^plus^. Furthermore, we hypothesized that their interaction with BC^plus^ would induce specific transcriptomic and metabolic signatures, revealing distinct solubilization strategies.

By combining genomic, transcriptomic, and physiological analyses under controlled conditions, this study aims to provide mechanistic insights into how PSB respond to and influence BC^plus^ solubilization, and to explore their role in mobilizing P from low-solubility fertilizers.

## Materials and methods

### Strain origins and properties

The bacterial strains were isolated in May 2016 from agricultural soils in Rostock, Germany (54°3′41.47″N, 12°5′5.59″E), which did not receive any inorganic P fertilizer since 1998 [19]. The plots were part of a controlled field trial that included different fertilization treatments, such as unfertilized controls and compost amendments.

For bacterial isolation, 50 g of topsoil was collected from the unfertilized control and the compost-amendment plot. PSB were isolated by serial dilution plating [21] on Pikovskaya agar supplemented with 10 gL^−1^ tricalcium phosphate (The Carl Roth GmbH + Co. KG, Germany) [22]. Plates were incubated at 28°C for 7 days under aerobic conditions until clear halo zones appeared, indicating phosphate solubilization. Colonies forming clear halos were screened based on halo size and distinct morphology to ensure functional and taxonomic diversity.

To capture potential differences in microbial traits shaped by long-term soil management, we selected one isolate from the unfertilized control plot (*Psychrobacillus sp.* INOP01) and one from the compost-amended plot (*B. licheniformis* COM1), both dominating the obtained isolates as well as showing strong and reproducible halo formation.

Additionally, *B. velezensis* DSM 23117, a well-characterized commercial bioinoculant with known PGP potential, was used as a reference strain. It was originally isolated from pathogen-suppressed soil in Germany [23, 24] and was obtained from the DSMZ culture collection (Leibniz Institute DSMZ). The genome of *B. licheniformis* COM1 was sequenced and assembled in this study (BioProject accession PRJNA1297992). The complete genome sequence of *P. psychrodurans* INOP01 has been published previously [20]. For *B. velezensis* DSM 23117, publicly available genome data were used (GenBank accession: CP000560).

### Genome analysis

Detailed workflows are provided in the Supplementary Methods.

### DNA extraction and sequencing

Genomic DNA from *B. licheniformis* was extracted using the Genomic-tip 20/G kit (Qiagen). Whole genome sequencing was performed using the PacBio Sequel SMRT platform.

### Assembly, annotation, and phylogenomic classification

Genomes were assembled HGAP4 (SMRT Link v9.0.0.92188), circularized (Circlator v1.5.5), quality checked (CheckM v1.1.2) and annotated (RAST v2.0). Phylogenomic placement was determined with TYGS, dDDH (GGDC 3.0), and FASTME v2.1.6.1 (see Table S2, S3).

### Functional gene identification

PGP-associated genes were identified using PLABase [25], focusing on phosphate solubilization, S metabolism, and phytohormone pathways (Table S4). Additional P-turnover genes, such as phosphate transporters, were screened manually (Table S5).

### Cultivation experiments

Detailed cultivation procedures, replicate structure, and statistical workflows are provided in the Supplementary Methods.

For all experiments, strains were precultured overnight at 30°C and 180 rpm in Belitzky Minimal Medium (BMM) supplemented with Full P (see Table S1). Cells were then harvested (3750 × g, 5 min), washed once with phosphate-buffered saline (PBS), and resuspended in BMM supplemented with either Full P (1 mM KH_2_PO_4_), P limitation (0.3 mM KH_2_PO_4_), or BC^plus^ (3.8 gL^−1^). The BC^plus^ concentration enables measurable solubilization while maintaining lower P availability than the P limitation treatment. It is consistent with previous studies [26, 27] and reflects ~3.3× field-equivalent loading [17], as higher doses are needed in cultivation systems to ensure sufficient mineral contact and overcome the absence of soil buffering.

**Figure 1:**
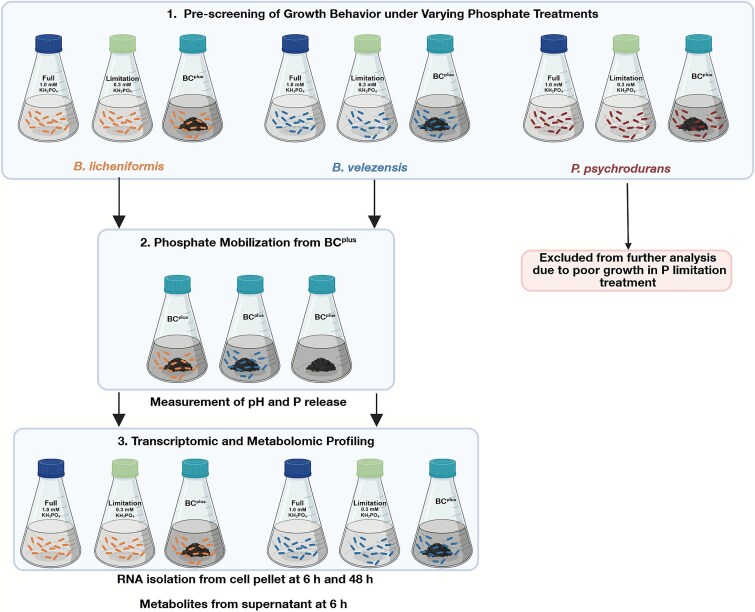
Overview of the experimental workflow. Three *Bacillus* strains were first screened for growth under different phosphate treatments (Full P, P limitation, BC^plus^). Based on poor growth performance under BC^plus^, *P. psychrodurans* was excluded from further analysis. *B. licheniformis* and *B. velezensis* were then used for two experiments: (i) phosphate mobilization assays with BC^plus^ as the sole phosphorus source, with soluble P and pH analyzed at 0, 3, 6, 24, 48, and 72 h; and (ii) transcriptomic and metabolomic profiling under three P treatments (Full P, P limitation, BC^plus^), with RNA isolated from cell pellets at 6 h and 48 h and metabolite sampling from the supernatant at 6 h*.*

To assess P-related phenotypic and molecular responses, a stepwise approach was used: growth screening under different P treatments, those with sufficient growth under all treatments were tested for solubilization of BC^plus^, followed by transcriptomic and metabolomic profiling (Fig. 1).

### Prescreening of growth behavior under varying phosphate treatments

Strains were cultured in BMM media described above (Full P, P limitation, and BC^plus^; Table S1). Cultures were inoculated at optical density at 500 nm (OD_500_) 0.05 and monitored over 10 h. OD_500_ was measured hourly over 10 h using a GENESYS 30 photometer (Thermo Scientific). Each treatment was tested in triplicate and repeated on two separate days. Based on these results, *P. psychrodurans* INOP01 was excluded from further analysis because it did not reach sufficient and consistent biomass under phosphate limitation, preventing inclusion in the full factorial design required for comparative transcriptomic and metabolomic profiling. The two *Bacillus* strains were selected because they showed reproducible growth across all treatments, enabling direct mechanistic comparison.

Growth curves were visualized in R v 4.3.1 [28] using LOESS smoothing with 95% confidence intervals.

### Phosphate mobilization from BC^plus^

*B. licheniformis* and *B. velezensis* were cultivated in BMM with BC^plus^ as the sole P source. Cultures were inoculated at OD_500_ of ~0.1 and incubated for 48 h. An uninoculated medium served as a control.

pH and soluble phosphate were monitored at several timepoints up to 72 h. Each treatment was tested with five biological replicates and repeated in two independent experimental runs conducted on separate days, with identical conditions across all treatments.

Statistical analyses were conducted in R v 4.3.1 [28] using generalized least squares models to assess strain and time effects, with post-hoc tests for pairwise comparisons.

### Transcriptomic analysis

Detailed protocols for sampling, RNA extraction, data processing, bioinformatics pipeline, including software parameters, workflow steps, read quality, and mapping rates and statistical analysis are provided in the Supplementary Methods.

*B. licheniformis* and *B. velezensis* were cultivated in BMM under Full P, P limitation, or BC^plus^ treatment at 30°C and 180 rpm for 48 h. Samples for transcriptomics were collected at two timepoints: when cultures reached OD_500_ of 0.4 (~6 h), and after 48 h. Metabolomic samples were collected only at 6 h.

### RNA extraction, transcriptome library preparation, and sequencing

Total RNA was extracted using a modified PureLink™ RNA Mini Kit (Invitrogen™) with additional TRIzol. RNA quality and integrity were assessed fluorometrically (Quant-iT™ RiboGreen® RNA Assay Kit; Thermo Fisher Scientific) and with a Fragment Analyzer5200 instrument (Agilent Technologies, Inc.). Ribosomal Integrity Number values were recorded for each sample.

cDNA synthesis (200 ng input) was done with the SuperScript™ IV VILO™ Mastermix (Invitrogen™). and library preparation was performed with NEBNext Ultra II FS DNA Library Prep Kit (New England BioLabs Inc.), followed by Illumina NextSeq 550 sequencing (NextSeq High Output Kit v2.5; 2 × 150 bp paired-end; ~3 M paired-end reads per sample).

### Read processing and mapping

Reads were quality-checked and trimmed, then pseudo-aligned with kallisto v0.51.0 to the annotated genomes of *B. velezensis* and *B. licheniformis*. Homologous sequences were identified with reciprocal best-hits mmseqs2 easy-rbh v13.45111. Noncoding and translation factor reads were removed. In total, 60 libraries were generated (2 strains × 3 treatments × 2 time points × 5 replicates); after excluding two low-quality samples, 58 remained, comprising a median of 1.9 × 10^5^ reads across 3655 annotated genes.

### Statistical analysis

Gene expression data were transformed into centered log ratios before statistical analysis. Singular Value Decomposition was applied to visualize sample clustering. Permutational Multivariate Analysis of Variance (PERMANOVA, vegan v2.6-4) was used to assess sample variance, and Generalized Linear Models were applied to identify significant predictors of gene expression. Pairwise contrasts between treatments were computed to determine condition-specific differential expression (see Supplementary Methods for details).

### Metabolite analysis

Metabolites in culture supernatants were quantified by LC–MS using a HILIC separation on a Sciex ExionLC AD coupled to a ZenoTOF 7600 system. Supernatants were prepared from the same cultures used for RNA-Seq, proteins were precipitated with acetonitrile, and five biological replicates per treatment were analyzed. Data processing and statistical analysis were performed in R. A detailed description of sample preparation, LC–MS settings, and metabolite annotation is provided in the Supplementary Information.

## Results

### Initial characterization of the strains

#### Genotypic characterization

Whole genome sequencing of the two bacterial isolates *B. licheniformis* and *Psychrobacillus psychrodurans* was performed to characterize their genomic potential for PGP trait. These were compared to a commercially available reference strain, *B. velezensis* DSM 23117.

Species identity was confirmed via digital DNA–DNA hybridization (dDDH), detailed in Table S3. *B. licheniformis* shows 97.4% similarity to the type strain DSM13, confirming its taxonomic classification. Its genome consists of a single circular chromosome (3.67 Mbp, 46.2% GC), encoding 3962 coding sequences (CDSs), 21 rRNAs, and 59 tRNAs, with 98.96% completeness and no detectable contamination (Table S2). Functional annotation using PLaBAse revealed the presence of all 21 conserved phosphate-solubilization genes (Fig. S1A). *B. licheniformis* encoded no unique genes in this category. It shared 10 S metabolism genes with *B. velezensis* that were absent in *P. psychrodurans* (Fig. S1B) and harbored one unique gene (*speA*) associated with phytohormone production (Fig. S1C). Manual curation of genes related to phosphorus turnover (Table S5) showed the presence of both low-affinity (*pit*) and high-affinity (*pst*) phosphate transporters, as well as *phoD* and *phy*, encoding alkaline phosphatase and phytase. Elements of the Pho regulon for gene regulation were also identified (Table S6).

*P. psychrodurans* shows only 36.3% dDDH similarity to the closest type strain (*P. psychrodurans* DSM 11713) (Table S3), suggesting that it represents a previously undescribed lineage within the genus [20]. Its genome comprises two contigs totaling 5.29 Mbp (35.5% GC), including a putative plasmid (213 kbp). It encodes 5757 CDSs, 37 rRNAs, and 111 tRNAs, with 99.34% completeness and 1.06% contamination (Table S2). PLaBAse analysis identified all 21 conserved phosphate-solubilization genes (Fig. S1A), and six unique ones (*pqqD, mqo, aceB|glcB, aceA, fumA|fumB, aldB*). It also encoded two unique S-related genes (*frc|yfdW* and *ssuE*, Fig. S1B) and one unique gene (*ispA*) linked to phytohormone synthesis (Fig. S1C). Gene screening (Table S5) confirmed *pit*, *pst*, and *appF* transporters. In addition to *phoD* and *phy*, *P. psychrodurans* carried *appA* (acid phosphatase/phytase) and multiple *phn* genes (e.g. *phnXW*, *phnOB*, *phnA*), involved in phosphonate degradation (Table S6). Pho regulon elements were present.

The genome of *B. velezensis* DSM 23117 consists of a single circular chromosome (3.92 Mbp, 46.4% GC), encoding 3799 CDSs, 29 rRNAs, and 89 tRNAs (Table S2). PLaBAse annotation revealed all 21 conserved phosphate-solubilization genes (Fig. S1A) and one unique gene (*prpE*). In S metabolism, *B. velezensis* shared 10 genes with *B. licheniformis* (Fig. S1B), while no unique phytohormone-related genes were detected (Fig. S1C). According to targeted screening (Tables S5, S6), it harbored *pit* and *pst* transporters, *phoD* and *phy*, and components of the Pho regulon, but lacked additional organic or phosphonate-associated genes.

### Strain performance under varied phosphorus treatments

All strains exhibited measurable growth when BC^plus^ was provided as the sole inorganic P source. For *B. licheniformis* and *B. velezensis*, growth rates were highest under BC^plus^, followed by Full P, and lowest under P limitation. *P. psychrodurans* grew under BC^plus^ and full P supply, but to a lower OD compared to the other strains and showed minimal growth under P limitation (Fig. S2).

Based on these phenotypic and genotypic insights, we selected *B. licheniformis* and *B. velezensis* for detailed mechanistic studies. *B. velezensis* (DSM 23117), used in commercial products by Abitep GmbH (https://abitep.de/) is well established as a biopesticide and biofertilizer [24, 29], while *B. licheniformis*, known for its PGP potential [30], is commercialized by Novobac and widely used in research and agriculture. The two strains also represent an ecologically meaningful contrast: *B. licheniformis*, isolated from compost, and *B. velezensis*, from nutrient-poor soil [24], both encode the full Pho regulon but differ in their gene copy numbers and P acquisition gene repertoires. These ecological and genomic differences make them suitable models for studying species-specific P adaptation strategies.

Although *P. psychrodurans* showed some growth under BC^plus^, its insufficient biomass under P limitation prevented reliable transcriptome or metabolite sampling. As our aim was to resolve condition-specific and time-resolved responses across all treatments, we focused on strains with robust and reproducible growth under all three conditions.

### Unraveling P solubilization mechanisms for BC^plus^

#### Strain selection and phenotypic validation of P solubilization

**Figure 2:**
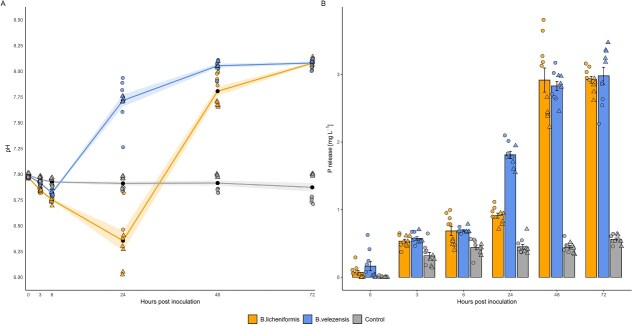
pH dynamics and P release over time in *B. licheniformis* and *B. velezensis*. (A) pH changes over 72 h post-inoculation in cultures of *B. licheniformis* (orange), *B. velezensis* (blue), and an uninoculated control (gray). Each point represents an individual measurement, with different point shapes indicating the two independent experimental runs. Black points show the mean values per time point, and colored lines represent the mean trends over time with shaded ribbons indicating standard error. (B) P release [mg l^−*1*^] is measured in the same cultures over time. Bars represent mean values, with individual data points shown. Error bars indicate standard deviation. Experiments were conducted on two separate days, with each treatment tested in five biological replicates per experiment (*n* = 10 per treatment in total).

To validate genomic predictions, *B. licheniformis* and *B. velezensis* were cultivated with BC^plus^ as sole P source and compared to an uninoculated control (Fig. 2). Both strains acidified the medium within 6 h (*B. licheniformis*: ~6.1 and for *B. velezensis*: ~ 6.6), while the control remained near pH 7.0.

After 72 h, both mobilized ~3 mg kg^−1^ of P, whereas the control showed minimal release. *B. velezensis* initiated solubilization earlier (from 3 h, *P* = .0151) than *B. licheniformis* (from 6 h, *P* = .0214). At 24 h, *B. velezensis* released significantly more P than the control (*P* < .0001) and outperformed *B. licheniformis* (*P* < .0001). The difference between the strains diminished at 48 and 72 h (*P* = .599 and *P* = .795, respectively), indicating a plateau in P mobilization (Tables S7–S8).

#### Transcriptomic characterization

**Figure 3:**
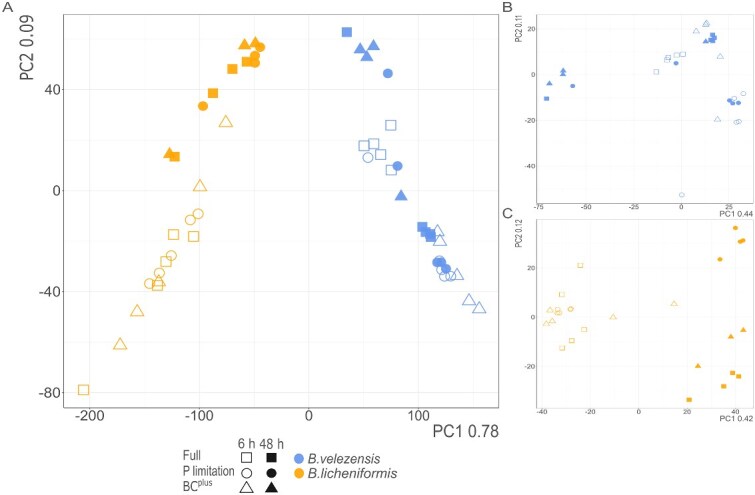
PCA of transcriptomic profiles in *B. velezensis* and *B. licheniformis* under different P treatment. (A) PCA plot of global transcriptional changes in *B. velezensis* (blue) and *B. licheniformis* (orange) grown in Full P (squares), P limitation (circles), and BC^plus^ (triangles) at 6 h (open symbols) and 48 h (filled symbols). (B) and (C) show separate PCAs for the strains, respectively. Each treatment was analyzed with five biological replicates per time point.

**Figure 4:**
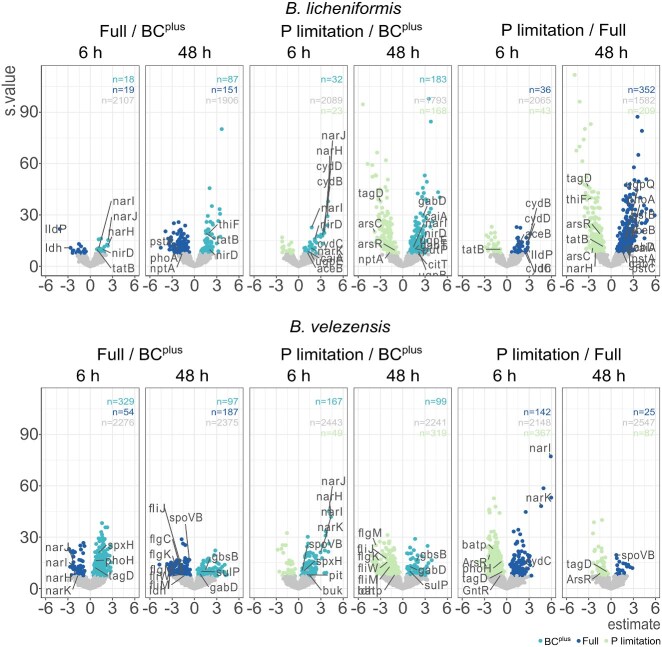
Differentially expressed genes (DEGs) in *B. licheniformis* and *B. velezensis* under different treatment and time points. The volcano plot shows the DEGs in *B. licheniformis* (top) and *B. velezensis* (bottom) under BC^plus^ vs Full P and BC^plus^ vs P limitation at 6 h and 48 h. Nonsignificant genes are shown in gray, while significant genes are colored according to the experimental treatments (BC^plus^, Full P, and P limitation). The x-axis represents the magnitude of expression change (estimate). The y-axis represents the statistical significance (s-value), with higher values indicating more significant changes in gene expression. Key genes of interest are labeled in the plot for easy identification.

**Figure 5:**
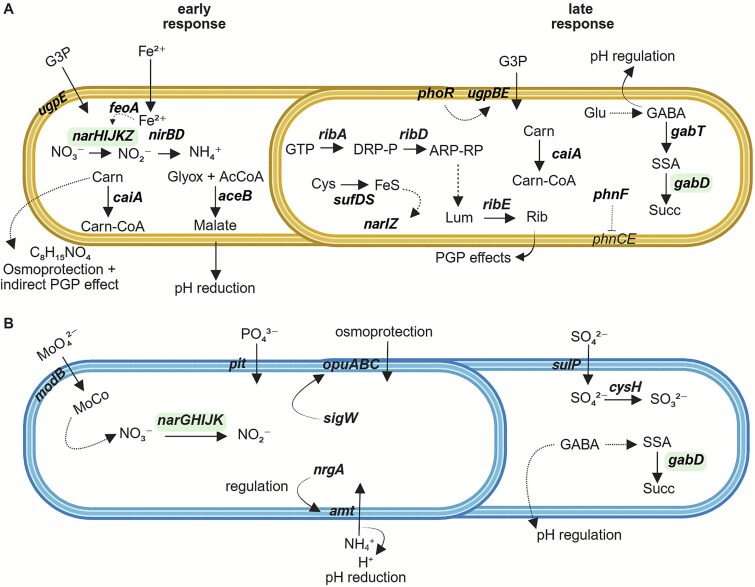
Schematic representation of differentially regulated genes in *Bacillus licheniformis* (A) and *Bacillus velezensis* (B) under BC^plus^ compared to P limitation. Gene expression was analyzed at 6 h (early) and 48 h (late) using RNA-seq. All genes shown were significantly upregulated under BC^plus^. Dashed arrows indicate inferred metabolic steps or regulatory interactions not directly captured at the transcriptional level. Bold italics highlight transcriptionally induced genes. Genes highlighted in light green were upregulated in both strains under BC^plus^, indicating shared responses. Abbreviations: BC^plus^, S enriched bone char fertilizer; G3P, glycerol-3-phosphate; Carn, L-carnitine; SSA, succinate semialdehyde; Succ, succinate; Lum, lumazine; rib, riboflavin; GABA, γ-aminobutyric acid; MoCo, molybdenum cofactor; PGP, plant-growth-promoting. (A) In *B. licheniformis*, early responses include G3P uptake (*ugpE*), ferrous iron import (*feoA*), nitrate respiration (*narHIJKZ, nirBD*), and malate formation via the glyoxylate shunt (*aceB*), contributing to acidification. *caiA*-mediated carnitine degradation and detection of a carnitine derivative. Riboflavin biosynthesis (*ribA, ribD, ribE*) proceeds via GTP-derived intermediates (DRP-P, ARP-RP, Lum), potentially supporting redox balance and plant interactions. The GABA shunt (*gabT, gabD*) contributes to pH regulation via glutamate-derived succinate formation. Regulatory genes (*phoR, phnF, ceiA*) may coordinate phosphate and carnitine responses. (B) In *B. velezensis*, early induction includes inorganic phosphate uptake (*pit*), ammonium assimilation (*amt, nrgA),* and nitrate respiration via *narGHJK*, which depends on MoCo biosynthesis via *modB*. The alternative sigma factor *sigW* and compatible solute transporter *opuABC* suggest osmotic stress adaptation. At 48 h, *sulP* and *cysH* mediate sulfate uptake and reduction, while *gabD* expression supports continued GABA shunt activity and pH regulation.

**Figure 6:**
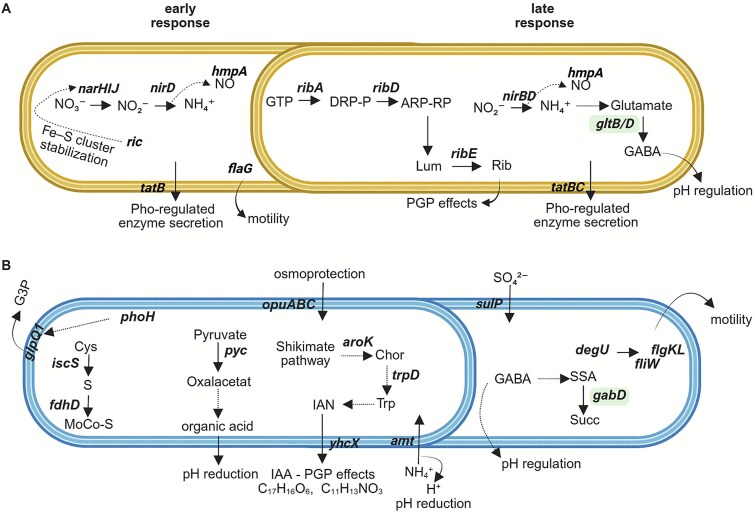
Schematic representation of differentially regulated genes in *Bacillus licheniformis* (A) and *Bacillus velezensis* (B) under BC^plus^ compared to Full P treatment. Gene expression was analyzed at 6 h (early) and 48 h (late) using RNA-seq. All genes shown were significantly upregulated under BC^plus^ relative to Full P. Dashed arrows indicate inferred metabolic steps or regulatory interactions not directly captured at the transcriptional level. Bold italics highlight transcriptionally induced genes. Genes highlighted in light green were upregulated in both strains under BC^plus^, indicating shared responses. Abbreviations: BC^plus^, S enriched bone char fertilizer; G3P, glycerol-3-phosphate; NO, nitric oxide; SSA, succinate semialdehyde; Succ, succinate; Rib, riboflavin; Lum, lumazine; Trp, tryptophan; IAN, indole-3-acetonitrile; IAA, indole-3-acetic acid; Chor, chorismite; GABA, γ-aminobutyric acid; MoCo, molybdenum cofactor; PGP, plant-growth-promoting. (A) In *B. licheniformis*, early responses include nitrate respiration (*narHIJ, nirD*), nitric oxide detoxification (*hmpA*), and Pho-regulated enzyme secretion (*tagBC*). Upregulation of *ric* suggests Fe–S cluster stabilization. *flgC* expression indicates enhanced motility. At 48 h, sustained *hmpA* activity and GABA biosynthesis via *gabT/gabD* support pH regulation. *ribA, ribD,* and ribE drive riboflavin biosynthesis via GTP-derived intermediates, with PGP effects. (B) In *B. velezensis*, early BC^plus^ responses involve P stravation (*phoH*), shikimate pathway (*aroK, trpD*), and indole compound biosynthesis (IAN, IAA). *pyc*-mediated oxaloacetate formation suggests increased organic acid production. MoCo biosynthesis (*iscS, fdhD*) and nitrate respiration (*narGHJ*) are supported by S and redox metabolism. At 48 h, upregulation of *gabD* suggests pH regulation, *sulP* indicates increased sulfate uptake, and the motility regulator *degU* along with downstream flagellar genes (*fligW, flgK, flgL*) suggests enhanced bacterial motility.

To evaluate the treatment-specific transcriptomic responses of *B. licheniformis* and *B. velezensis*, RNA-Seq-based differential gene expression analyses were conducted at 6 and 48 hours for three treatments: Full P, P limitation, and BC^plus^. Principal component analysis (PCA) showed clear separation between the two species, with strain identity explaining ~69% of variance (R^2^ = 0.692, *P* < .001; Fig. 3). Time point (4.3%; R^2^ = 0.043, *P* = .002) and P treatment (1.9%; R^2^ = 0.019, *P* = .039) contributed less. PERMANOVA therefore was performed separately for each species. In *B. licheniformis*, variance was mainly explained by time (19.7%; R^2^ = 0.197, *P* < .001), followed by treatment (11.6%; R^2^ = 0.116, *P* = .007) and their interaction (10.2%; R^2^ = 0.102, *P* = .012). In *B. velezensis*, treatment was the strongest factor (15.6%; R^2^ = 0.156, *P* = .006), followed by interaction (13.9%; R^2^ = 0.139, *P* = .006) and time (10.9%; R^2^ = 0.109, *P* = .002). Thus, strain identity was the primary driver of transcriptional differences, while the relative influence of P condition and time varied between species. Transcriptional responses were therefore analyzed separately for *B. licheniformis* and *B. velezensis* across both time points (Figs 4–6). Figure 4 summarizes the global differential expression patterns, while Figures 5 and 6 highlight BC^plus^ induced pathways relative to P limitation and Full P, respectively.

At 6 h, *B. licheniformis* showed a slow transcriptional response to BC^plus^, with 32 genes upregulated relative to P limitation, including *ugpE* (glycerol-3-P uptake), *narKIJHZ*, *nirBD* (nitrate/nitrite reduction), *aceB* (malate synthase), *feoA* (ferrous iron transporter), and *hmpA* (nitrosative stress). Simultaneously, 23 genes were downregulated, among them *capA*, *capC*, *pgsB* (exopolysaccharide biosynthesis), *murB*, *pel* (cell wall metabolism), and *phoR*, *ykaA* (phosphate regulation). Similar patterns were observed versus Full P medium, with 19 genes upregulated in BC^plus^, including *narHIJ*, *nirD*, and *hmpA* and 19 genes were downregulated, including *glpR* (glycerol regulon repressor).

Several genes were consistently upregulated in BC^plus^ compared to both P limitation and Full P, including *sufDS*, *iscA* (Fe–S cluster biosynthesis), *prpB*, *prpD* (organic acid catabolism), *gabD*, *gabT* (GABA shunt), and *opuBBCCBA* (osmoprotection). Additional genes included *dhbC* (bacillibactin biosynthesis), *ribD*, *ribE* (riboflavin biosynthesis), *mmgD* (malate metabolism), *nirB/D*, *corA* (Mg^2+^ transport), *peptT* (peptide transport), and *gltB/D* (glutamate synthase).

After 48 h, BC^plus^ induced 183 genes compared to P limitation, including *cysI* (S metabolism), *narI*, *ugpB/E*, *phnF*, *phoR* (P turnover), *citT* (citrate/succinate transport), *putP* (proline transport), *betB* (glycine betaine synthesis), and *ribA* (riboflavin biosynthesis). Meanwhile, 168 genes were downregulated under BC^plus^, including *lytR, tagD, murB, pel* (cell wall metabolism), *phoR, ykaA* (phosphate regulation), *secA (protein secretion), ptsG, ptsH* (PTS system), *arsC, arsR* (arsenate response), *spxA, gloA* (oxidative stress), *abrB, spo0E, spoVG* (sporulation), *sigI* (alternative sigma factor), and *tatB* (protein export). In comparison to Full P, BC^plus^ induced fewer genes, including *phoH2* (P stress), but 151 were downregulated, including *murB* and *murG* (cell wall synthesis), and *ldh* and *lldP* (lactate metabolism). A shared set of genes downregulated in both BC^plus^ and P limitation (vs. Full P) included *cotD*, *sleL*, *spoIIE*, *sigE/G* (sporulation), *pstABC*, *phoA* (P uptake), and *nagAB*, *glmM* (amino sugar/peptidoglycan biosynthesis).

In contrast to BC^plus^, P limitation triggered a distinct transcriptional profile. Compared to Full P, 43 genes were upregulated, including *tatB* (protein export), *gerD* (sporulation), *lanM* (lantibiotic modification), *hpaE*, *dmpH* (aromatic compound degradation), and 36 were downregulated compared to Full P treatment, including *glpD* (glycerol-3-phosphate dehydrogenase), *ptsN* (PTS component), *yutH* (sporulation), and *aceB*. At 48 h, P limitation triggered upregulation of 209 genes compared to Full P, many related to nitrate reduction (*narH*, *narZ*), protein secretion (*secG*), stress (*arsC/R*, *spxA*, *gloA*), and sporulation (*spo0E*, *spoVG*, *sigI*). At this time point, 358 genes were downregulated in P limitation compared to Full P treatment, including sporulation genes (*gerAA/AB/AC*), cell wall metabolism (*murQ*, *cwlJ*), and organic acid metabolism (*mmgD*, *aceA/B*, *prpB/D*).

In contrast to *B. licheniformis*, *B. velezensis* showed a broader and earlier transcriptional response to BC^plus^. At 6 h, 167 genes were upregulated in BC^plus^ relative to P limitation, including genes involved in organic acid metabolism (*paas*, *pyc*, *accC*), IAA precursor biosynthesis (*trpD*), organic P mobilization (*glpQ1*), stress response regulation (*phoH*, *spxH*), *nadE* (NAD^+^ biosynthesis), *ftsW* (cell division), and *wcaA* (capsule biosynthesis). Several genes related to N metabolism and nitrate respiration were significantly induced, including those associated with nitrate transporter (*narK*) and ammonium transporter (*nrgA*), as well as respiratory nitrate reductase subunits (*narG*HIJ). Conversely, 49 genes were downregulated compared to P limitation, including those involved in secretion (*secG*, *exdp*), amino acid metabolism (*mrp*, *lrp*, *dat*, *odhB*), carbohydrate processing (*galE*, *dctP*), and envelope regulation (*pgsB*, *mecA*, *yajQ*). Relative to Full P treatment, 329 genes were upregulated in BC^plus^ at 6 h, including genes such as *nadE*, *ftsW*, and *wcaA*. 53 genes were downregulated in BC^plus^ compared to Full P, including *lutA* (lactate metabolism), *tatC* (protein export), *modAB* (molybdate transport), and *spoIIIAE* (sporulation).

By 48 h, fewer genes responded. Ninety-nine genes were upregulated in BC^plus^ compared to P limitation, including *uvrA* (DNA repair), *pyro* (pyruvate metabolism), and *cysh* (S metabolism). In contrast, 319 genes were downregulated, particularly those related to motility (*fliS*, *fliD*, *flgL/K/P/M*, *fliW*, *motAB*), organic acid metabolism (*pyc*), nutrient transport (*ptsH*, *ptsN*), and regulatory genes (*sigI*, *spoVT*, *spo0J*, *yabP*, *phoP*, *phoB1*). Compared to Full P at 48 h, 97 genes were upregulated in BC^plus^, including *acoAB* (acetoin metabolism), *gabD* (GABA metabolism), *gbsB* (glycine betaine synthesis), and *hydH* (redox regulation). 187 genes were downregulated, including genes associated with flagellar assembly (*flgI/K/M*, *fliW*, *fliM*), purine biosynthesis (*purQ/N/D*), and secondary metabolite synthesis (*pksJ/N*, *bacG/D/C*). Also, *spo0J* (sporulation regulator) was repressed.

Beyond the BC^plus^ response, comparison of P limitation to Full P revealed additional regulatory patterns. At 6 h, 367 genes were upregulated in P limitation, including those involved in cell wall and membrane biosynthesis (*tagD*, *murQ*), oxidative stress defense (*ahpC/F*), sporulation and stress regulation (*sigH*, *sigI*, *spo0J*, *yabQ*), phosphate metabolism (*phoH*), carbohydrate transport (*ptsG*, *oppC*), and motility (*cheC*). Additional genes included *aroK* (aromatic amino acid biosynthesis), *arsR* (metal resistance), *gntR* (transcriptional regulator), and *sdhC* (respiratory chain). One hundred forty-two genes were downregulated compared to Full P, including *lutB* and *lutA* (lactate utilization), *narG* (nitrate reductase), *gatA* (galactitol metabolism), *tatC* (protein export), *modAB* (molybdate transport), and *spoIIIAE* (sporulation). At 48 h, 87 genes were upregulated in P limitation compared to Full P, such as *tagD*, *aroK*, and *arsR*, while 25 were downregulated, including *spo0J*.

### Metabolomic profiling

At 6 h, extracellular metabolite profiles showed clear treatment- and species-specific differences (putative annotations). *B. velezensis* produced a broader metabolite range under BC^plus^ (94 features in positive mode, 2 in negative) compared to *B. licheniformis* (13 and 4, respectively), indicating overall higher metabolic activity. Several metabolites aligned with transcriptomic patterns (Fig. S4).

In *B. velezensis*, N-acetyl-α-muramate was detected under BC^plus^, consistent with the upregulation of cell wall biosynthesis genes (*murQ*, *murB*, *flgK*) observed in this treatment. The detection of aromatic compounds with molecular formulas C_11_H_13_NO_3_ and C_17_H_16_O_6_ coincided with upregulation of genes such as *aroK* and *trpD*, involved in aromatic amino acid biosynthesis and the shikimate pathway. Additionally, butyrylglycine, a short-chain fatty acid conjugate, was detected and matched the increased expression of *buk* and other genes involved in organic acid metabolism.

In *B. licheniformis*, a carnitine-related compound (C_8_H_15_NO_4_) was observed in BC^plus^ treatment. This corresponds to the upregulation of *caiA*, a gene associated with carnitine metabolism.

In addition to these treatment-specific compounds, 2-amino-5-hydroxyheptanedioic acid in *B. velezensis* and a feature annotated as C_15_H_22_N_6_O in *B. licheniformis* were consistently enriched under both BC^plus^ contrasts.

## Discussion

Our results show that BC^plus^, despite its relatively low P solubility, supported bacterial growth as the sole P source. This confirms its potential as a P input for microbial systems and low-input agriculture. Growth under BC^plus^ even exceeded full inorganic P supply, suggesting benefits beyond P, likely due to S compounds and micronutrients enhancing microbial activity. This is consistent with observations from biochar-amended soils, where S and P mobilizers promoted plant growth [31].

Our comparative transcriptomic and metabolomic analysis of *B. velezensis* and *B. licheniformis* reveals species-specific strategies for phosphate solubilization, metabolic regulation, and stress response. These differences show that phenotypic similarity in solubilization can mask substantial mechanistic divergence and highlight strain-level routes to a shared outcome, underscoring the need for a holistic perspective on inoculant design. The contrasting strategies likely reflect differences in ecological origin and adaptive history, which are explored in more detail below.

### Strain-specific responses under P limitation and full P availability

Phosphate scarcity drives bacterial adaptation via the PhoPR system [32], which may reflect regulatory divergence or specialization to organic or microbially modified phosphate sources. Functional decoupling of *phoPR* from phosphate sensing has been shown in *Mycobacterium tuberculosis*, where PhoPR instead responds to acidic pH and salt stress [33, 34]. In *Streptomyces*, PhoP activity varies despite conserved genes, as lower PhoPR protein abundance in *S. coelicolor* versus *S. lividans* affects central metabolism and antibiotic production [35]. Additionally, variability in PHO box structure and promoter context can reduce or abolish PhoP binding [36, 37]. These findings show that *phoPR* presence alone does not ensure a canonical phosphate starvation response and may explain the lack of functional activation in *P. psychrodurans*.

In *B. velezensis*, phosphate limitation triggered a rapid and multi-layered response: early *phoH* activation indicated Pho regulon induction, accompanied by *tagD* upregulation, suggesting cell wall remodeling under phosphate stress [38–41], along with stress-related genes like *arsR* and *batP*. Notably, *arsR* was upregulated despite the absence of arsenic, likely due to chemical mimicry between phosphate and arsenate, which can activate *ars* genes via phosphate transporters [42]. Upregulation of *batP*, suggests secondary metabolism is also responsive to nutrient stress, a common trait in *Bacillus* species adapted to oligotrophic environments [43]. These features likely reflect the oligotrophic origin of *B. velezensis*, favoring fast and plastic adaptation to fluctuating nutrient availability. In contrast, *B. licheniformis* displayed a delayed and more gradual adaptation. Early *tatB* induction suggests early stress perception, possibly tied to Pho-regulated enzyme secretion [44, 45]. However, Pho-related genes like *phoH2*, a P-stress-associated helicase/nuclease [46–48], and *ykaA*, a *phoU*-like gene [49] were only induced after 48 h, alongside sporulation markers, indicating a delayed, survival-oriented response [50].

Under Full P treatment, both species shifted away from classical PhoPR-regulated stress responses and toward nutrient utilization and secondary metabolism. *B. licheniformis* showed signs of carbon overflow (upregulation of glycerol metabolism, N assimilation), suggesting excess phosphate induced carbon flux redirection [51]. Concurrent upregulation of *phnF*, a repressor of phosphonate transport, suggests that cells sensed internal P sufficiency and actively suppressed alternative acquisition route [52]. Interestingly, low-affinity phosphate transporter *nptA*, a low-affinity phosphate transporter typically induced under P limitation [53] remained expressed, possibly due to localized P depletion near the cell surface caused by high uptake rates. Additional *glnA* induction indicates N-P co-regulation [54].

*B. velezensis*, in contrast, activated PGP-associated and N assimilation pathways already at 6 h, including *budA* and *alsS*, and nitrate reduction genes. This suggests tight coupling between P and N metabolism even under nonlimiting conditions [55, 56]. By 48 h, *B. velezensis* showed signs of stress anticipation and increased motility, marked by upregulation of flagellar genes and the sporulation markers. Similar Pho-regulated flagellar responses have been reported in *Vibrio cholerae* under P limitation [57]. Concurrent induction of secondary metabolism genes (*pks*, *bac*) suggests early activation of biosynthetic pathways, potentially linked to plant interaction or microbial competition.

### Strain-specific P mobilization strategies

Although BC^plus^ contains high total P (107 g kg^−1^), only ~0.7% is water-soluble [15], making initial availability low. Both species successfully mobilized P from BC^plus^, but via distinct strategies, underscoring their functional divergence.

In *B. licheniformis*, early *aceB* (6 h) and later *citT* (48 h) induction, together with a pH minimum at 24 h (Fig. 1A), suggest a gradual trajectory driven by malate-associated organic acid production, consistent with organic acid-mediated solubilization [58]. The delayed induction of *phoR*, indicates a late phosphate starvation response. Persistent *ugpE* expression and subsequent *ugpB* induction support uptake of glycerophosphodiesters—organic phosphate forms usually repressed under high intracellular P [59, 60]. *phnF* induction, together with increased soluble phosphate at 48 h (Fig. 1B), may indicate partial alleviation of phosphate stress [52]. In contrast, *B. velezensis* showed a faster, more dynamic response, with >300 DEGs and ~90 significantly enriched metabolites under BC^plus^ treatment. At 6 h, *pyc* (oxaloacetate-linked acidification) [60] was already upregulated, alongside *phoH* (P limitation marker [33]) and the phosphate transporter *pit*. These early shifts paralleled rapid phosphate release by 24 h (Fig. 1B). By 48 h, acidification genes had declined, while *gabD* contributed to succinate formation via the GABA shunt, supporting pH regulation and redox balance [61]. Consistent with its stronger transcriptional activation, *B. velezensis* also secreted a markedly higher number of extracellular metabolites under BC^plus^ compared to *B. licheniformis* (Fig. S4). This aligns with the significantly higher number of BC^plus^ responsive metabolites secreted by *B. velezensis*. This broader and faster metabolic response of *B. velezensis*, marked by a significantly higher number of secreted metabolites, highlights its greater plasticity and likely reflects adaptation to more variable ecological niches.

### Co-regulation of non-P mobilizing genes in the presence of BC^plus^

#### Nutrient cross-regulation: coupling of P, N, and S metabolism

In bacteria, P, N, and S are tightly interconnected through energy metabolism, cofactor synthesis, and redox balance [62]. Under BC^plus^ both strains upregulated *nar* operon genes and nitrate/nitrite transporters, likely reflecting redox imbalance rather than oxygen limitation [63, 64]. The heterogeneous composition of BC^plus^ may also cause nutrient imbalance and suboptimal uptake of key elements.

In *B. licheniformis*, this was accompanied by strong induction of *fdhD*, which supplies S to molybdenum cofactors and supports dissimilatory nitrate reduction to ammonium (DNRA). This suggests sequential activation of the nitrate reduction pathway and highlights the integration of P, N, and S metabolism under phosphate-limited, S-rich conditions [65, 66]. Co-upregulation of *narI* and *sufDS* further supports coordination between Fe–S cluster maturation and nitrate respiration, while induction of *hmpA* and *ric* indicates activation of oxidative and nitrosative stress responses [67, 68].

In *B. velezensis*, early *amt* (ammonium transporter) induction may reflect PhoPR cross-regulation, contributing to acidification and P solubilization [69, 70]. Unlike the cofactor-linked response of *B. licheniformis*, *B. velezensis* displayed a broader S stress program, with induction of *sulP* (S transporter), *spxH* (redox regulator), and *yrk* (sulfide detoxification) [71, 72]. Similar P–S cross-regulation has been observed in *Microcystis aeruginosa* and *Bacillus subtilis* under phosphate stress [73, 74], but was absent under P limitation alone, suggesting that the high S content (3.2 g kg^−1^) of BC^plus^ specifically amplifies this response.

The earlier induction of S stress and transport genes in *B. velezensis*, compared to the more targeted cofactor-related response in *B. licheniformis*, suggests higher metabolic readiness and may explain the faster transcriptomic adjustment of *B. velezensis* under BC^plus^ conditions.

At 48 h, strain responses diverged. In *B. licheniformis*, genes for nitrate respiration (*nar*, *nir*), nitric oxide detoxification (*hmpA*), Fe–S cluster assembly (*suf*), and S carrier proteins (*thiS/F*) remained elevated, consistent with reports in *Pantoea dispersa* and *Pseudomonas aeruginosa*, where Fe–S biosynthesis supports phosphate solubilization under stress [75]. In contrast, *B. velezensis* had downregulated nitrate reductases but maintained *sulP* expression, suggesting resolution of N stress but continued S acquisition.

#### Stress mitigation and osmoprotection

Although both *Bacillus* species solubilized P from BC^plus^, the two *Bacillus* employed distinct stress responses, underscoring functional divergence behind an apparently redundant trait. Both upregulated proline and compatible solute transporters, with earlier activation in *B. velezensis*, consistent with known osmotic and acid stress responses in *B. megaterium* [13]. Sustained *gabD* expression indicates activation of the glutamate decarboxylase pathway, which buffers cytosolic pH under acid stress [76–79]. This is supported by the consistent accumulation of 2-amino-5-hydroxyheptanedioic acid under BC^plus^, a compound likely derived from lysine degradation via 5-aminovalerate and glutarate, as described in *Escherichia coli* [80].

*B. licheniformis* upregulated *caiA* and produced a carnitine derivative C_8_H_15_NO_4_ [81], suggesting osmoprotection under acid stress, similar to *Pseudomonas fluorescens* [82]. Carnitine functions as a compatible solute under acid and osmotic stress [83, 84], and exogenous carnitine can promote barley germination under salt stress [85], pointing to a potential indirect role in plant–microbe interaction. Additionally, a consistently enriched N rich feature annotated as C_15_H_22_N_6_O is consistent with a peptide-based osmolyte or redox-active metabolite. Bacteria frequently accumulate small peptides or amino acid derivatives to buffer osmotic or oxidative stress, especially under nutrient imbalance [86–88].

*B. velezensis* additionally showed signs of active cell wall remodeling. Upregulation of *mur* genes and accumulation of N-acetyl-α-muramate suggest peptidoglycan turnover [89, 90], while *glpQ* expression indicates phosphate scavenging from cell wall teichoic acids [91]. Detection of butyrylglycine together with *buk* expression suggests rerouting of butyrate for detoxification, as described in *Bacillus subtilis* under acid stress [92].

### Induction of plant-growth-promoting traits

Beyond P solubilization, both strains expressed distinct PGP traits, illustrating how similar outcomes can emerge from different regulatory strategies. Although isolated from bulk soil, *B. licheniformis* is often found in crop rhizospheres [93, 94], supporting its plant-associated potential. At 48 h, upregulation of *ribA*, *ribD*, and *ribE* (riboflavin biosynthesis) indicated increased riboflavin production, known to promote root growth and systemic resistance at low concentrations. Late *gabT* induction, involved in GABA synthesis, may further reflect emerging plant-interactive capacity, as GABA contributes to acid tolerance and auxin-mediated nutrient uptake [61].

In contrast, *B. velezensis* showed an earlier, broader PGP signature. Early *aroK* induction (6 h) and detection of aromatic compounds (C_17_H_16_O_6_ and C_11_H_13_NO_3_) point to activation of the shikimate pathway, which provides precursors for aromatic amino acids and secondary metabolites [95]. This pathway is linked to phosphate solubilization and rhizosphere competence in *Bacillus* spp. [90], potentially supporting flavonoid-like signaling. Both plants and microbes modulate flavonoid and phenylpropanoid biosynthesis during interaction [96], and some bacteria may directly produce flavonoid-like molecules [97]. Concurrent upregulation of *trpD* and *yhcX*, involved in indole-3-acetic acid (IAA) biosynthesis, suggests early auxin-related activity, consistent with known IAA production in *Azospirillum* and *Bacillus* [98, 99]. Bacterial IAA enhances root development and nutrient uptake, improving plant resilience to abiotic stress [100]. Together, these findings suggest that multi-element fertilizers like BC^plus^ can stimulate broader microbial activity beyond P solubilization.

Taken together, these results highlight that similar functions can emerge from distinct regulatory programs without implying ecological redundancy, emphasizing the role of niche history in shaping microbial responses to complex P fertilizers.

## Conclusions

This study reveals strain-specific strategies that lead to convergent phosphate-solubilization outcomes in *Bacillus.* While both strains successfully mobilized P from BC^plus^, their transcriptional and metabolic responses differed: *B. velezensis* rapidly activated acidification, nitrate respiration, detoxification, and PGP traits, while *B. licheniformis* showed a slower trajectory with DNRA, Fe–S cluster biosynthesis, and delayed induction of plant-interactive traits, likely reflecting ecological origins and regulatory flexibility.

These patterns emerged under BC^plus^ treatment, a complex inorganic P substrate. While initially triggering phosphate starvation responses, BC^plus^ also induced broader metabolic programs. Its low immediate P availability demands microbial solubilization, while micronutrients support sustained activity.

Our multi-omics analysis reveals tightly linked P, N, and S metabolism, with species-specific co-activation of redox regulation, cofactor synthesis, and stress buffering, underscoring the need for inoculant development that combines complementary, strain-specific pathways shaped by niche history.

Overall, these findings support pairing alternative P fertilizers like BC^plus^ with tailored PSB strains. Such combinations may enhance nutrient cycling, plant productivity, and ecological fit. Future studies should validate these findings in the field and test whether consortia can exploit functional divergence to improve performance.

## Supplementary Material

03_Supplementary_material_ycaf208

## Data Availability

The complete genome sequences of the strains have been deposited in GenBank under accession numbers CP073315, CP093291, CP093292, CP093293, and CP093290. The raw sequencing data have been deposited in the NCBI Sequence Read Archive (SRA) under the BioProject PRJNA1327818. The dataset is currently under embargo and will be publicly released upon acceptance of the manuscript. For peer review, the data can be accessed via the following confidential reviewer link: https://dataview.ncbi.nlm.nih.gov/object/PRJNA1327818?reviewer=u8j6drms39j6o2itl5gfeekr6m. All scripts and code used for transcriptomic data processing, statistical analysis, and figure generation are available at https://github.com/rsiani/thaqi_2025. Strains will be deposited at the Leibniz Institute DSMZ (Braunschweig, Germany).

## References

[1] Hedley MJ, Stewart JWB, Chauhan BS. Changes in inorganic and organic soil phosphorus fractions induced by cultivation practices and by laboratory incubations. *Soil Sci Soc Am J* 1982;46:970–6. 10.2136/sssaj1982.03615995004600050017x

[2] Schoumans OF, Bouraoui F, Kabbe C et al. Phosphorus management in Europe in a changing world. *Ambio* 2015;44:180–92. 10.1007/s13280-014-0613-9 PMC4329153 25681976

[3] Mogollón JM, Beusen AHW, van Grinsven HJM et al. Future agricultural phosphorus demand according to the shared socioeconomic pathways. *Glob Environ Chang* 2018;50:149–63. 10.1016/j.gloenvcha.2018.03.007

[4] Alewell C, Ringeval B, Ballabio C et al. Global phosphorus shortage will be aggravated by soil erosion. *Nat Commun* 2020;11:4546. 10.1038/s41467-020-18326-7 32917863 PMC7486398

[5] Jokkaew S, Jantharadej K, Pokhum C et al. Free and encapsulated phosphate-solubilizing bacteria for the enhanced dissolution of swine wastewater-derived struvite—an attractive approach for green phosphorus fertilizer. *Sustainability* 2022;14:12627. 10.3390/su142012627

[6] Magallon-Servin P, Antoun H, Taktek S et al. Designing a multi-species inoculant of phosphate rock-solubilizing bacteria compatible with arbuscular mycorrhizae for plant growth promotion in low-P soil amended with PR. *Biol Fertil Soils* 2020;56:521–36. 10.1007/s00374-019-01418-3

[7] Mohd Din ARJ, Rosli MA, Mohamad Azam Z et al. *Paenibacillus polymyxa* role involved in phosphate solubilization and growth promotion of *Zea mays* under abiotic stress condition. *Proc Natl Acad Sci India Sect B Biol Sci* 2020;90:63–71. 10.1007/s40011-019-01127-y

[8] Nosratabad ARF, Etesami H, Shariati S. Integrated use of organic fertilizer and bacterial inoculant improves phosphorus use efficiency in wheat (*Triticum aestivum* L.) fertilized with triple superphosphate. *Rhizosphere* 2017;3:109–11. 10.1016/j.rhisph.2017.04.004

[9] Illmer P, Schinner F. Solubilization of inorganic calcium phosphates—solubilization mechanisms. *Soil Biol Biochem* 1995;27:257–63. 10.1016/0038-0717(94)00190-C

[10] Sharma S, Sayyed R, Trivedi M et al. Phosphate solubilizing microbes: sustainable approach for managing phosphorus deficiency in agricultural soils. *Springerplus* 2013;2:587. 10.1186/2193-1801-2-587 25674415 PMC4320215

[11] Wani PA, Khan MS, Zaidi A. Synergistic effects of the inoculation with nitrogen-fixing and phosphate-solubilizing rhizobacteria on the performance of field-grown chickpea. *J Plant Nutr Soil Sci* 2007;170:283–7. 10.1002/jpln.200620602

[12] Chen Y, Rekha P, Arun A et al. Phosphate solubilizing bacteria from subtropical soil and their tricalcium phosphate solubilizing abilities. *Appl Soil Ecol* 2006;34:33–41. 10.1016/j.apsoil.2005.12.002

[13] Fan X, Habib L, Fleckenstein J et al. In situ digestion: A concept to manage soil phosphate in organic farming. In: Proceedings of 13th International Fertilizer Symposium: Fertilizers in Context with Resource Management in Agriculture. Turkey: Tokat, 2002.

[14] Fan X, Ewald S, Silvia H et al. In situ digestion of rock phosphates to mobilize plant-available phosphate for organic farming. *Commun Soil Sci Plant Anal* 2012;43:2191–201. 10.1080/00103624.2012.708073

[15] Zimmer D, Panten K, Frank M et al. Sulfur-enriched bone char as alternative P fertilizer: spectroscopic, wet chemical, and yield response evaluation. *Agriculture* 2019;9:21. 10.3390/agriculture9010021

[16] Thaqi SK, Siani R, Chiba A et al. Effects of novel P fertilizers on microbial abundance related to N and P cycling in two on-farm systems. *Agric Ecosyst Environ* 2025;385:109565. 10.1016/j.agee.2025.109565

[17] Kruse J, Panten K, Siebers N. The fate of phosphorus from bone char-based fertilizers in soil pools in a 5-year crop rotation. *Nutr Cycl Agroecosyst* 2022;124:263–77. 10.1007/s10705-022-10228-y

[18] Grafe M, Kurth JK, Panten K et al. Effects of different innovative bone char based P fertilizers on bacteria catalyzing P turnover in agricultural soils. *Agric Ecosyst Environ* 2021;314:107419. 10.1016/j.agee.2021.107419

[19] Peine M, Vitow N, Grafe M et al. Effect of triple superphosphate and biowaste compost on mycorrhizal colonization and enzymatic P mobilization under maize in a long-term field experiment. *J Plant Nutr Soil Sci* 2019;182:167–74. 10.1002/jpln.201800499

[20] Chiba A, Peine M, Kublik S et al. Complete genome sequence of *Psychrobacillus* sp. strain INOP01, a phosphate-solubilizing bacterium isolated from an agricultural soil in Germany. *Microbiol Resour Announc* 2022;11:e00207–22. 10.1128/mra.00207-22 35377163 PMC9022493

[21] Somasegaran P, Hoben H. Handbook for Rhizobia Methods in Legume-Rhizobium Technology. New York: Springer, 1994.

[22] Pikovskaya R . Mobilization of phosphorus in soil in connection with the vital activity of some microbial species. *Mikrobiologiya* 1948;17:362–70.

[23] Krebs B, Höding B, Kübart S et al. Use of *Bacillus subtilis* as biocontrol agent. I. Activities and characterization of *Bacillus subtilis* strains. *J Plant Dis Prot* 1998;105:181–97.

[24] Chen XH, Koumoutsi A, Scholz R et al. Comparative analysis of the complete genome sequence of the plant growth–promoting bacterium *bacillus amyloliquefaciens* FZB42. *Nat Biotechnol* 2007;25:1007–14. 10.1038/nbt1325 17704766

[25] Patz S, Gautam A, Becker M et al. PLaBAse: a comprehensive web resource for analyzing the plant growth-promoting potential of plant-associated bacteria. *bioRxiv* 2021;2021.12.13.472471. 10.1101/2021.12.13.472471

[26] Saeid A, Labuda M, Chojnacka K et al. Valorization of bones to liquid phosphorus fertilizer by microbial solubilization. *Waste Biomass Valoriz* 2014;5:265–72. 10.1007/s12649-013-9235-8

[27] Wyciszkiewicz M, Saeid A, Chojnacka K et al. Production of phosphate biofertilizers from bones by phosphate-solubilizing bacterium *Bacillus megaterium*. *Open Chem* 2015;13:1–10. 10.1515/chem-2015-0123

[28] R Core Team . R: A Language and Environment for Statistical Computing. Vienna: R Foundation for Statistical Computing, 2023.

[29] Fan B, Wang C, Song X et al. *Bacillus velezensis* FZB42 in 2018: the gram-positive model strain for plant growth promotion and biocontrol. *Front Microbiol* 2018;9:2491. 10.3389/fmicb.2018.02491 30386322 PMC6198173

[30] Bashan Y, Moreno M, Troyo E. Growth promotion of the seawater-irrigated oilseed halophyte *Salicornia bigelovii* inoculated with mangrove rhizosphere bacteria and halotolerant *Azospirillum* spp. *Biol Fertil Soils* 2000;32:265–72. 10.1007/s003740000246

[31] Fierer N, Bradford MA, Jackson RB. Toward an ecological classification of soil bacteria. *Ecology* 2007;88:1354–64. 10.1890/05-1839 17601128

[32] Hulett FM, Lee J, Shi L et al. Sequential action of two-component genetic switches regulates the PHO regulon in *Bacillus subtilis*. *J Bacteriol* 1994;176:1348–58. 10.1128/jb.176.5.1348-1358.1994 8113174 PMC205199

[33] Singh PR, Goar H, Paul P et al. Dual functioning by the PhoR sensor is a key determinant to *mycobacterium tuberculosis* virulence. *PLoS Genet* 2023;19:e1011070. 10.1371/journal.pgen.1011070 38100394 PMC10723718

[34] Ryndak M, Wang S, Smith I. PhoP, a key player in *mycobacterium tuberculosis* virulence. *Trends Microbiol* 2008;16:528–34. 10.1016/j.tim.2008.08.006 18835713

[35] Millan-Oropeza A, Henry C, Lejeune C et al. Expression of genes of the pho regulon is altered in *Streptomyces coelicolor*. *Sci Rep* 2020;10:8492. 10.1038/s41598-020-65087-w 32444655 PMC7244524

[36] Martín JF, Santos-Beneit F, Rodríguez-García A et al. Transcriptomic studies of phosphate control of primary and secondary metabolism in *Streptomyces coelicolor*. *Appl Microbiol Biotechnol* 2012;95:61–75. 10.1007/s00253-012-4129-6 22622839

[37] Allenby NEE, Laing E, Bucca G et al. Diverse control of metabolism and other cellular processes in *Streptomyces coelicolor* by the PhoP transcription factor: genome-wide identification of in vivo targets. *Nucleic Acids Res* 2012;40:9543–56. 10.1093/nar/gks766 22904076 PMC3479208

[38] Bhavsar AP, Erdman LK, Schertzer JW et al. Teichoic acid is an essential polymer in *Bacillus subtilis* that is functionally distinct from teichuronic acid. *J Bacteriol* 2004;186:7865–73. 10.1128/jb.186.23.7865-7873.2004 15547257 PMC529093

[39] Stosiek N, Talma M, Klimek-Ochab M. Carbon–phosphorus lyase—the state of the art. *Appl Biochem Biotechnol* 2020;190:1525–52. 10.1007/s12010-019-03161-4 31792787

[40] Ellwood DC, Tempest DW. Control of teichoic acid and teichuronic acid biosyntheses in chemostat cultures of *Bacillus subtilis* var. niger. *Biochem J* 1969;111:1–5. 10.1042/bj1110001 4975313 PMC1187486

[41] Lang WK, Glassey K, Archibald AR. Influence of phosphate supply on teichoic acid and teichuronic acid content of *Bacillus subtilis* cell walls. *J Bacteriol* 1982;151:367–75. 10.1128/jb.151.1.367-375.1982 6806244 PMC220249

[42] Wang Q, Kang YS, Alowaifeer A et al. Phosphate starvation response controls genes required to synthesize the phosphate analog arsenate. *Environ Microbiol* 2018;20:1782–93. 10.1111/1462-2920.14108 29575522

[43] Haavik HI . Studies on the formation of bacitracin by *bacillus licheniformis*: effect of inorganic phosphate. *Microbiology* 1974;84:226–30. 10.1099/00221287-84-1-226 4436646

[44] Eder S, Shi L, Jensen K et al. A *Bacillus subtilis* secreted phosphodiesterase/alkaline phosphatase is the product of a pho regulon gene, phoD. *Microbiology* 1996;142:2041–7. 10.1099/13500872-142-8-2041 8760916

[45] Jongbloed JDH, Martin U, Antelmann H et al. TatC is a specificity determinant for protein secretion via the twin-arginine translocation pathway. *J Biol Chem* 2000;275:41350–7. 10.1074/jbc.M004887200 11007775

[46] Caldwell R, Sapolsky R, Weyler W et al. Correlation between *Bacillus subtilis* scoC phenotype and gene expression determined using microarrays for transcriptome analysis. *J Bacteriol* 2001;183:7329–40. 10.1128/jb.183.24.7329-7340.2001 11717292 PMC95582

[47] Mäder U, Homuth G, Scharf C et al. Transcriptome and proteome analysis of *Bacillus subtilis* gene expression modulated by amino acid availability. *J Bacteriol* 2002;184:4288–95. 10.1128/jb.184.15.4288-4295.2002 12107147 PMC135197

[48] Shivangi KY, Ekka MK et al. Structural and functional characterization of mycobacterial PhoH2 and identification of potential inhibitor of its enzymatic activity. *Braz J Microbiol* 2024;55:1033–51. 10.1007/s42770-024-01267-4 38386260 PMC11153397

[49] Molle V, Fujita M, Jensen ST et al. The Spo0A regulon of *Bacillus subtilis*. *Mol Microbiol* 2003;50:1683–701. 10.1046/j.1365-2958.2003.03818.x 14651647

[50] Jensen KK, Sharkova E, Duggan MF et al. *Bacillus subtilis* transcription regulator Spo0A decreases alkaline phosphatase levels induced by phosphate starvation. *J Bacteriol* 1993;175:3749–56. 10.1128/jb.175.12.3749-3756.1993 8509330 PMC204791

[51] Kleijn RJ, Buescher JM, Le Chat L et al. Metabolic fluxes during strong carbon catabolite repression by malate in *Bacillus subtilis*. *J Biol Chem* 2010;285:1587–96. 10.1074/jbc.M109.061747 19917605 PMC2804316

[52] Gebhard S, Busby JN, Fritz G et al. Crystal structure of PhnF, a GntR-family transcriptional regulator of phosphate transport in *mycobacterium smegmatis*. *J Bacteriol* 2014;196:3472–81. 10.1128/jb.01965-14 25049090 PMC4187667

[53] Kelliher JL, Radin JN, Grim KP et al. Acquisition of the phosphate transporter NptA enhances *Staphylococcus aureus* pathogenesis by improving phosphate uptake in divergent environments. *Infect Immun* 2018;86:e00631–17. 10.1128/iai.00631-17 PMC5736819 29084897

[54] Sola-Landa A, Rodríguez-García A, Amin R et al. Competition between the GlnR and PhoP regulators for the *glnA* and *amtB* promoters in *Streptomyces coelicolor*. *Nucleic Acids Res* 2013;41:1767–82. 10.1093/nar/gks1203 23248009 PMC3561978

[55] Russo A, Pollastri S, Ruocco M et al. Volatile organic compounds in the interaction between plants and beneficial microorganisms. *J Plant Interact* 2022;17:840–52. 10.1080/17429145.2022.2107243

[56] Huang Y, Chen J, Jiang Q et al. The molybdate-binding protein ModA is required for *Proteus mirabilis*-induced UTI. *Front Microbiol* 2023;14:1156273. 10.3389/fmicb.2023.1156273 37180242 PMC10174112

[57] Pratt JT, McDonough E, Camilli A. PhoB regulates motility, biofilms, and cyclic di-GMP in *vibrio cholerae*. *J Bacteriol* 2009;191:6632–42. 10.1128/jb.00708-09 19734314 PMC2795287

[58] Rawat P, Das S, Shankhdhar D et al. Phosphate-solubilizing microorganisms: mechanism and their role in phosphate solubilization and uptake. *J Soil Sci Plant Nutr* 2021;21:49–68. 10.1007/s42729-020-00342-7

[59] Brzoska P, Rimmele M, Brzostek K et al. The pho regulon-dependent Ugp uptake system for glycerol-3-phosphate in *Escherichia coli* is trans inhibited by pi. *J Bacteriol* 1994;176:15–20. 10.1128/jb.176.1.15-20.1994 8282692 PMC205009

[60] Yang K, Wang M, Metcalf WW. Uptake of glycerol-2-phosphate via the ugp-encoded transporter in *Escherichia coli* K-12. *J Bacteriol* 2009;191:4667–70. 10.1128/jb.00235-09 19429609 PMC2704724

[61] Smith J, Johnson A. Effects of *bacillus velezensis* FX-6 on GABA production and biomass increase in tomato plants. *J Agric Sci* 2021;18:350–65.

[62] Santos-Beneit F . The pho regulon: a huge regulatory network in bacteria. *Front Microbiol* 2015;6:402. 10.3389/fmicb.2015.00402 25983732 PMC4415409

[63] Goldová J, Ulrych A, Hercík K et al. A eukaryotic-type signalling system of *Pseudomonas aeruginosa* contributes to oxidative stress resistance, intracellular survival and virulence. *BMC Genomics* 2011;12:437. 10.1186/1471-2164-12-437 21880152 PMC3224232

[64] Martín JF, Rodríguez-García A, Liras P. The master regulator PhoP coordinates phosphate and nitrogen metabolism, respiration, cell differentiation and antibiotic biosynthesis: comparison in *Streptomyces coelicolor* and *streptomyces avermitilis*. *J Antibiot* 2017;70:534–41. 10.1038/ja.2017.19 28293039

[65] Liu S, Dai J, Wei H et al. Dissimilatory nitrate reduction to ammonium (DNRA) and denitrification pathways are leveraged by cyclic AMP receptor protein (CRP) paralogues based on electron donor/acceptor limitation in *Shewanella loihica* PV-4. *Appl Environ Microbiol* 2021;87:e01964–20. 10.1128/AEM.01964-20 33158888 PMC7783327

[66] Shao B, Niu L, Xie Y-G et al. Overlooked in-situ sulfur disproportionation fuels dissimilatory nitrate reduction to ammonium in sulfur-based system: novel insight of nitrogen recovery. *Water Res* 2024;257:121700. 10.1016/j.watres.2024.121700 38705068

[67] Yokoyama N, Nonaka C, Ohashi Y et al. Distinct roles for U-type proteins in iron–sulfur cluster biosynthesis revealed by genetic analysis of the *Bacillus subtilis* sufCDSUB operon. *Mol Microbiol* 2018;107:688–703. 10.1111/mmi.13907 29292548

[68] Porrini C, Guérin C, Tran S-L et al. Implication of a key region of six *Bacillus cereus* genes involved in siroheme synthesis, nitrite reductase production and iron cluster repair in the bacterial response to nitric oxide stress. *Int J Mol Sci* 2021;22:5079. 10.3390/ijms22095079 34064887 PMC8151001

[69] Wang Y, Cen XF, Zhao GP et al. Characterization of a new GlnR binding box in the promoter of *amtB* in *Streptomyces coelicolor* inferred a PhoP/GlnR competitive binding mechanism for transcriptional regulation of *amtB*. *J Bacteriol* 2012;194:5237–44. 10.1128/jb.00989-12 22821977 PMC3457235

[70] Lu Z, He S, Kashif M et al. Effect of ammonium stress on phosphorus solubilization of a novel marine mangrove microorganism *Bacillus aryabhattai* NM1-A2 as revealed by integrated omics analysis. *BMC Genomics* 2023;24:550. 10.1186/s12864-023-09559-z 37723472 PMC10506230

[71] Choi S-Y, Reyes D, Leelakriangsak M et al. The global regulator Spx functions in the control of organosulfur metabolism in *Bacillus subtilis*. *J Bacteriol* 2006;188:5741–51. 10.1128/jb.00443-06 16885442 PMC1540065

[72] Tang C, Li J, Shen Y et al. A sulfide-sensor and a sulfane sulfur-sensor collectively regulate sulfur-oxidation for feather degradation by *Bacillus licheniformis*. *Commun Biol* 2023;6:167. 10.1038/s42003-023-04538-2 36765168 PMC9918477

[73] Harke MJ, Gobler CJ. Global transcriptional responses of the toxic cyanobacterium, *Microcystis aeruginosa*, to nitrogen stress, phosphorus stress, and growth on organic matter. *PLoS One* 2013;8:e69834. 10.1371/journal.pone.0069834 23894552 PMC3720943

[74] Botella E, Hübner S, Hokamp K et al. Cell envelope gene expression in phosphate-limited *Bacillus subtilis* cells. *Microbiology* 2011;157:2470–84. 10.1099/mic.0.049205-0 21636651

[75] Priya P, Aneesh B, Sivakumar KC et al. Comparative proteomic analysis of saline tolerant, phosphate solubilizing endophytic *Pantoea* sp., and *pseudomonas* sp. isolated from *Eichhornia* rhizosphere. *Microbiol Res* 2022;265:127217. 10.1016/j.micres.2022.127217 36206648

[76] Feehily C, Karatzas KAG. Role of glutamate metabolism in bacterial responses towards acid and other stresses. *J Appl Microbiol* 2013;114:11–24. 10.1111/j.1365-2672.2012.05434.x 22924898

[77] Damiano MA, Bastianelli D, Dahouk SA et al. Glutamate decarboxylase-dependent acid resistance in *brucella* spp.: distribution and contribution to fitness under extremely acidic conditions. *Appl Environ Microbiol* 2015;81:578–86. 10.1128/AEM.02928-14 25381237 PMC4277594

[78] Feehily C, Finnerty A, Casey PG et al. Divergent evolution of the activity and regulation of the glutamate decarboxylase systems in *listeria monocytogenes* EGD-e and 10403S: roles in virulence and acid tolerance. *PLoS One* 2014;9:e112649. 10.1371/journal.pone.0112649 25386947 PMC4227838

[79] Su MS, Schlicht S, Gänzle MG. Contribution of glutamate decarboxylase in *lactobacillus reuteri* to acid resistance and persistence in sourdough fermentation. *Microb Cell Factories* 2011;10:S8. 10.1186/1475-2859-10-S1-S8 PMC3231934 21995488

[80] Knorr S, Sinn M, Galetskiy D et al. Widespread bacterial lysine degradation proceeding via glutarate and L-2-hydroxyglutarate. *Nat Commun* 2018;9:5071. 10.1038/s41467-018-07563-6 30498244 PMC6265302

[81] National Center for Biotechnology Information (NCBI) . PubChem Compound Summary for CID 71728466, Hydroxyoctadecadienylcarnitine. Bethesda, MD, USA: NCBI; 2025. Available at: https://pubchem.ncbi.nlm.nih.gov/compound/Hydroxyoctadecadienylcarnitine (accessed 2025.)

[82] MacLean A, Legendre F, Tharmalingam S et al. Phosphate stress triggers the conversion of glycerol into L-carnitine in *Pseudomonas fluorescens*. *Microbiol Res* 2021;253:126865. 10.1016/j.micres.2021.126865 34562839

[83] Meng X-L, Gao X, Si Y-M et al. Role of carnitine in adaptation of *Chromohalobacter salexigens* DSM 3043 and its mutants to osmotic and temperature stress in defined medium. *Extremophiles* 2022;26:28. 10.1007/s00792-022-01276-x 35964293

[84] Dykes GA, Moorhead SM. Survival of osmotic and acid stress by *listeria monocytogenes* strains of clinical or meat origin. *Int J Food Microbiol* 2000;56:161–6. 10.1016/S0168-1605(99)00205-6 10857542

[85] Oney-Birol S . Exogenous L-carnitine promotes plant growth and cell division by mitigating genotoxic damage of salt stress. *Sci Rep* 2019;9:17229. 10.1038/s41598-019-53542-2 31754247 PMC6872569

[86] Wu J, Yu Y, Liu F et al. γ-Aminobutyric acid (GABA) metabolic bypass plays a crucial role in stress tolerance and biofilm formation in *Cronobacter sakazakii* ATCC 29544. *Foods* 2025;14:171. 10.3390/foods14020171 39856838 PMC11764851

[87] Zaprasis A, Brill J, Thüring M et al. Osmoprotection of *Bacillus subtilis* through import and proteolysis of proline-containing peptides. *Appl Environ Microbiol* 2013;79:576–87. 10.1128/aem.01934-12 23144141 PMC3553765

[88] Sleator RD, Hill C. Bacterial osmoadaptation: the role of osmolytes in bacterial stress and virulence. *FEMS Microbiol Rev* 2002;26:49–71. 10.1111/j.1574-6976.2002.tb00598.x 12007642

[89] Vollmer W, Blanot D, De Pedro MA. Peptidoglycan structure and architecture. *FEMS Microbiol Rev* 2008;32:149–67. 10.1111/j.1574-6976.2007.00094.x 18194336

[90] Yang W, Zhao Y, Yang Y et al. A genomic analysis of *bacillus megaterium* HT517 reveals the genetic basis of its abilities to promote growth and control disease in greenhouse tomato. *Int J Genomics* 2022;2022:2093029. 10.1155/2022/2093029 36605453 PMC9810399

[91] Mayer C, Kluj RM, Mühleck M et al. Bacteria's different ways to recycle their own cell wall. *Int J Med Microbiol* 2019;309:151326. 10.1016/j.ijmm.2019.06.006 31296364

[92] Han SE, Kim KY, Maung CEH. *Bacillus subtilis* PE7-mediated alleviation of phosphate starvation and growth promotion of netted melon *Cucumis melo* L. *var reticulatus Naud Microorganisms* 2024;12:2384. 10.3390/microorganisms12232384 39770587 PMC11678189

[93] de O , de MFHV, de OTS et al. *Bacillus subtilis* and *bacillus licheniformis* promote tomato growth. Braz. *J Microbiol* 2023;54:397–406. 10.1007/s42770-022-00874-3 PMC9943921 36422850

[94] Cao H, Peng T, Zhao W et al. Whole-genome sequencing uncovers the plant growth-promoting potential of *bacillus licheniformis* G41, isolated from the rhizosphere soil of Gannan navel orange. *Ann Microbiol* 2025;75:8. 10.1186/s13213-025-01797-8

[95] Wu S, Chen W, Lu S et al. Metabolic engineering of shikimic acid biosynthesis pathway for the production of shikimic acid and its branched products in microorganisms: advances and prospects. *Molecules* 2022;27:4779. 10.3390/molecules27154779 35897952 PMC9332510

[96] Pang F, Solanki MK, Xing Y-X et al. *Streptomyces* improves sugarcane drought tolerance by enhancing phenylalanine biosynthesis and optimizing the rhizosphere environment. *Plant Physiol Biochem* 2024;217:109236. 10.1016/j.plaphy.2024.109236 39481196

[97] Okoye CO, Jiang H, Wu Y et al. Bacterial biosynthesis of flavonoids: overview, current biotechnology applications, challenges, and prospects. *J Cell Physiol* 2024;239:e31006. 10.1002/jcp.31006 37025076

[98] Zimmer W, Aparicio C, Elmerich C. Relationship between tryptophan biosynthesis and indole-3-acetic acid production in *Azospirillum*: identification and sequencing of a *trpGDC* cluster. *Mol Gen Genet* 1991;229:41–51. 10.1007/BF00264211 1896020

[99] Deng C, Liang X, Zhang N et al. Molecular mechanisms of plant growth promotion for methylotrophic *bacillus aryabhattai* LAD. *Front Microbiol* 2022;13:917382. 10.3389/fmicb.2022.917382 36353455 PMC9637944

[100] Etesami H, Glick BR. Bacterial indole-3-acetic acid: a key regulator for plant growth, plant–microbe interactions, and agricultural adaptive resilience. *Microbiol Res* 2024;281:127602. 10.1016/j.micres.2024.127602 38228017

