# Supplementary material for: Strain-specific strategies underlie convergent phosphate solubilization in Bacillus
Source: ISME Commun. 2025 Nov 14;5(1):ycaf208. doi: 10.1093/ismeco/ycaf208 (PMC12676729; doi:10.1093/ismeco/ycaf208)
Supplement: 03_Supplementary_material_ycaf208 [file 03_supplementary_material_ycaf208.docx]

**Supplementary material**

**Strain-specific strategies underlie convergent phosphate solubilization in *Bacillus***

**Stefanie** **Katharina Thaqi**, Roberto Siani, Akane Chiba, Manuela Peine, Christel Baum, Michael Witting, Susanne Walch, Peter Leinweber, Michael Schloter, Stefanie Schulz

**Corresponding author:** Stefanie Katharina Thaqi; stefanie.thaqi@tum.de

**Cultivation Experiments**

Strains were routinely maintained in Nutrient Broth medium (Carl Roth GmbH, Karlsruhe, Germany) and stored in 50% glycerol at –80 °C for long-term preservation. Before each experiment, strains were cultured overnight in with full P availability with 30 °C and 180 rpm. Before each experiment, strains were grown overnight at 30 °C and 180 rpm in Belitzky Minimal Medium (BMM) supplemented with Full P (see Table S1). BMM is a defined medium commonly used in *Bacillus* studies to simulate P limitation or enable nutrient-specific manipulation [1]. On the following day, cells were harvested (3,750 × g, 5 min), washed once with sterile phosphate-buffered saline (PBS), and resuspended in 1 mL of the respective test medium: BMM, a defined medium composed of inorganic salts, glucose, and amino acids (Table S1), supplemented with either Full P (1 mM KH_2_PO_4_), P limitation (0.3 mM KH_2_PO_4_), or BC^plus^ (3.8 g L^-1^).

To characterize P-related phenotypic and molecular responses, a stepwise approach was followed: All strains were first screened for growth under different P conditions (see Table S1), those with sufficient growth were tested for solubilization of BC^plus^, followed by transcriptomic and metabolomic profiling.

## **Pre-screening of Growth Behavior under Varying Phosphate Conditions**

Strains were cultured in BMM media described above (Full P, P limitation, and BC^plus^; see Table S1 for details on P source and concentrations). Cultures were inoculated at optical density at 500 nm (OD_500_) 0.05 into 25 mL medium in 100 mL Erlenmeyer flasks. OD_500_ was measured hourly over 10 hours using a GENESYS 30 photometer (Thermo Scientific). All treatments were tested in three biological replicates, and each experiment was independently repeated on two separate days, resulting in six replicates per treatment. Based on these results, *P. psychrodurans* INOP01 was excluded from further analysis due to its limited growth response under the P limitation treatment.

Growth curves were visualized in R v 4.3.1 [2] using the ggplot2 package for plotting . Smoothed growth trends were fitted using local polynomial regression (LOESS) with 95% confidence intervals.

**Phosphate Mobilization from BC^plus^**

To investigate the ability of selected strains to mobilize P from BC^plus^, *B. licheniformis* and *B. velezensis* were cultivated in 150 mL BMM supplemented with 3.8 gL^-1^ BC^plus^ as the sole P source. Cultures were inoculated to an initial OD_500_ of ~0.1 and incubated in 500 mL Erlenmeyer flasks at 30 °C and 180 rpm for 48 hours. An uninoculated medium served as a control.

The pH and soluble phosphate were measured at 0, 3, 6, and subsequently at 24, 48, and 72 hours. At each time point, 10 mL of culture was sampled, centrifuged (3,750 × g, 5 min), and the supernatant sterile filtered (0.22 µm, Millipore, USA). Soluble phosphate was quantified using the NANOCOLOR Orthophosphate Kit (Macherey-Nagel, Germany), and pH was determined with a basic pH meter (Sartorius, Germany). All experiments were performed with five biological replicates and repeated on two independent days, resulting in ten replicates per treatment.

Statistical analyses were conducted in R v 4.3.1 [2] using the nlme v3.1-162, emmeans v1.10.4, and dplyr v1.1.4 packages. A generalized least squares (GLS) model was fitted to evaluate the effects of strain and time on P release. The model included strain, time, and their interaction as fixed effects. An autoregressive correlation structure accounted for repeated measures within each biological replicate. Independent experiment days were represented by the factor bioReplicate and included in the correlation structure. Each strain–replicate combination was treated as a separate time series. An ANOVA assessed significance, and Tukey-adjusted post-hoc tests identified pairwise differences (p < 0.05).

**Table S1** Components of the BMM. A basic medium was initially prepared and then supplemented with different P sources to create three variations: Full supplement (1 mM KH₂PO₄, 31 mg total P L^-1^ and 95 mg plant-available P L^-1^), P limitation (0.3 mM KH₂PO₄, 9.3 mg total P L^-1^ and 28.5 mg plant-available), and BC^plus^ (3.8 g L^-1^ BC^plus^, 384 mg total P L^-1^, 4.1 mg plant-available P L^-1^, based on resin-P and NaHCO₃-extractable P fractions from [3]).

| **Components** | **Concentration** |
| --- | --- |
| (NH_4_)_2_SO_4_ | 15 mM |
| MgSO_4_ | 8 mM |
| KCl | 27 mM |
| Na_3_C_6_H_5_O_7_ | 7 mM |
| Tris | 50 mM |
| CaCl₂ 2 H_2_O | 2 mM |
| FeSO₄ 7 H_2_O | 10 µM |
| MnSO₄ H_2_O | 10 µM |
| L-Glutamic acid | 4.5 mM |
| D-Glucose | 0.20% |
| L-Tryptophan | 39 µM |
| P source | Varied |

**Bioinformatics Pipeline for Genome Analysis**

**Quality Control and Read Processing**

Genomic DNA was extracted from overnight cultures using the Genomic-tip 20/G kit (Qiagen) or an alternative SDS/NaClO_4_–chloroform/ethanol extraction. DNA fragmentation to approximately 10 kb was performed using g-TUBEs (Covaris, USA). Library preparation was carried out with the SMRTbell Express Template Prep Kit 2.0, and sequencing was conducted on the PacBio Sequel platform. The sequencing protocol included 120 minutes of immobilization, 120 minutes of pre-extension, and 600 minutes of movie time per SMRT cell.

**Demultiplexing and Read Quality**

After demultiplexing, between 33,916 and 131,882 polymerase reads were obtained, with an average subread length ranging from 3,201 to 4,420 bp.

**Read Processing, De Novo Assembly, and Genome Annotation**

The genomes of isolates were sequenced using the PacBio Sequel SMRT platform. The sequencing generated 33,916 reads for *Bacillus licheniformis* with a mean length of 3,313 bp (total: 1.82 billion bases, 375× coverage) and 80,545 reads for *Psychrobacillus psychrodurans* with a mean length of 4,420 bp (total: 4.09 billion bases, 895× coverage).

High-quality de novo genome assemblies were performed using the Hierarchical Genome-Assembly Process 4 (HGAP4) pipeline embedded in SMRT Link v9.0.0.92188 with default parameters. Circularization of chromosome scaffolds was performed using Circlator v1.5.5 (Hunt et al., 2015) with the parameter merge_min_length_merge 1000. The quality of the recovered microbial genomes was assessed using CheckM v1.1.2 (Parks et al., 2015), which estimated genome completeness and contamination. The final genome assemblies measured 4,301,978 bp for *Bacillus licheniformis* and 4,243,588 bp for *P. psychrodurans*, with GC contents ranging from 35.54% to 46.20%. Each strain contained a single circular chromosome, except for *P. psychrodurans*, which included two additional contigs, potentially plasmids and contained 4,311, 5,757 and 3,962 predicted coding sequences (CDSs), 74, 111, 59 tRNA-coding genes, and 30, 37, 21 rRNA genes, respectively. (Table S2). The genome of the commercially available strain *B. velezensis* FZB42 contained 3,693 coding sequences (CDSs), a GC content of 46.4%, 89 tRNA genes, and 10 rRNA genes [4].

**Identification of Closely Related Reference Strains and Phylogenomic Inference**

The identification of closely related type strains was performed using the Type Strain Genome Server (TYGS) (Meier-Kolthoff and Göker, 2019) following a two-step approach.

In the first step, target genomes were compared against all type strain genomes available in the TYGS database using the MASH algorithm (Ondov et al., 2016). The ten type strains with the smallest MASH distances were selected for each target genome.

In the second step, a 16S rDNA-based analysis was conducted. The 16S rDNA gene sequences were extracted from the target genomes using RNAmmer (Lagesen et al., 2007) and subsequently BLASTed (Camacho et al., 2009) against 16S rDNA sequences from 19,307 type strains in the TYGS database. The top 50 matches (based on BLAST bitscore) were selected. Intergenomic distances were then calculated using Genome BLAST Distance Phylogeny (GBDP) with distance formula d_5_ (Meier-Kolthoff et al., 2013) under the 'coverage' algorithm. Based on these distances, the ten closest type strain genomes were identified.

**Phylogenomic Inference and Digital DNA-DNA Hybridization (dDDH) Analysis**

Digital DNA-DNA hybridization (dDDH) values and confidence intervals were calculated using the Genome-to-Genome Distance Calculator (GGDC) 3.0 (Meier-Kolthoff et al., 2022) with recommended settings. A minimum evolution tree was constructed from the intergenomic distances using FASTME v2.1.6.1 (Lefort et al., 2015), employing subtree pruning and regrafting (SPR) for postprocessing. Branch support values were determined through 100 pseudo-bootstrap replicates. The resulting phylogenetic tree was midpoint-rooted (Farris, 1972) and visualized using PhyD3 (Kreft et al., 2017).

For target strains with a maximum dDDH value below the 70% species threshold (Goris et al., 2007), an extended phylogenetic analysis based on 16S rRNA gene sequences was performed. For target strains with a maximum dDDH value below the 70% species threshold (Goris et al., 2007), an extended phylogenetic analysis based on 16S rRNA gene sequences was performed. Phylogenies were inferred using the GGDC web server (Meier-Kolthoff et al., 2022) through the DSMZ phylogenomic pipeline (Meier-Kolthoff et al., 2014), which was adapted for single-gene analysis. Multiple sequence alignments were generated with MUSCLE (Edgar, 2004). Phylogenetic trees were constructed using two approaches. Maximum Likelihood (ML) trees were inferred with RAxML (Stamatakis, 2014) using rapid bootstrapping combined with the autoMRE bootstopping criterion (Pattengale et al., 2010), followed by a search for the best-scoring tree. Maximum Parsimony (MP) trees were generated with TNT (Goloboff et al., 2008), applying 1,000 bootstrap replicates, tree-bisection-and-reconnection (TBR) branch swapping, and ten random sequence addition replicates. To assess sequence composition, a compositional bias test was performed using the Χ² test, as implemented in PAUP* (Swofford and Sullivan, 2003). The genomes were annotated for functional categories and biological subsystems using Rapid Annotations based on Subsystems Technology (RAST) v2.0 (Aziz et al., 2008).

The species identities of the isolates were determined through whole-genome comparisons using the TYGS server. For the P. psychrodurans isolate, the closest match was Psychrobacillus psychrodurans DSM 11713, with a G+C content difference of 0.41% and a dDDH value of 36.3%, indicating that it represents a distinct species. For the B. licheniformis isolate, the closest type strain was Bacillus licheniformis DSM 13, with a G+C content difference of 0.02% and a dDDH value of 97.4%, confirming species identity (Table S3).

**Functional Gene Identification**

PGP-associated genes were identified using PLABase [26], a curated trait-based annotation resource. Genes annotated as “direct effects” (trait1 level) were extracted, and categories related to phosphate solubilization, S metabolism, and phytohormone production (trait2/3 levels) were analyzed. The full gene set used for trait annotation and Venn diagram construction is listed in Table S4. In addition, we manually screened the genomes for a broader set of functional genes involved in microbial P turnover, beyond the scope of PLABase trait categories, like genes related to inorganic phosphate transport. The curated list of screened P-related genes is provided in Table S5.

**Transcriptome Analysis**

**Experimental Design and Sampling**

To investigate cellular and molecular mechanisms responses to different P sources, *B. licheniformis* and *B. velezensis* were cultivated in 150 mL BMM under three conditions (full P, P limitation, and BC^plus^) 30°C and 180 rpm for 48 h. Cultures were inoculated at an initial OD_500_ of ~0.1 in five biological replicates. Samples for transcriptomic analysis were taken at two time points: when cultures reached OD_500_ of 0.4 (corresponding to ~6 h), and after 48 h. Metabolomic samples were collected only at 6 h. At each time point, cultures were centrifuged (3,750 × g, 5 min); volumes were adjusted to yield approximately 10⁹ cells per sample. Cell pellets were snap-frozen in liquid nitrogen and stored at -80 °C for transcriptomics, and supernatants (6 h only) were sterile-filtered (0.22 µm, Millipore, USA) and stored at -80 °C for metabolomic analysis.

**RNA Extraction, Transcriptome Library Preparation, and Sequencing**

Total RNA was extracted using a modified protocol based on the PureLink^TM^ RNA Mini Kit (Invitrogen^TM^) in combination with TRIzol reagent. For cell lysis, pellets were first resuspended in a lysozyme solution and incubated for 45 minutes at 37 °C. This was followed by mechanical disruption using Precellys24 (Bertin Technologies) bead-beating at full speed for over 45 seconds. Column-based DNase digestion was omitted during the PureLink protocol. Instead, residual genomic DNA was enzymatically removed post-extraction, and samples were subsequently cleaned using the RNA Clean & Concentrator-5 Kit (Zymo Research). To confirm the absence of genomic DNA in RNA samples, PCR targeting the 16S rRNA gene was performed using the universal bacterial primers 27f (5'-AGAGTTTGATCMTGGCTCAG-3') and 1492r (5'-GGTTACCTTGTTACGACTT-3') (metabion international AG). The PCR reaction was carried out in a 25 µL volume consisting of 16.25 µL PCR-grade water, 2.5 µL 10× buffer (Invitrogen), 0.5 µL dNTPs (10 mM, Thermo Scientific), 1.5 µL MgCl_2_ (25 mM, Invitrogen), 2.5 µL BSA (20 mg/mL), 0.5 µL of each primer (10 pmol/µL), 0.25–0.35 µL Taq Polymerase (5 U/µL, Invitrogen), and 0.5 µL RNA sample as template. Thermal cycling was performed with an initial denaturation at 94 °C for 10 minutes, followed by 35 cycles of 94 °C for 1 minute, 56 °C for 1 minute, and 72 °C for 2 minutes. A final extension step was carried out at 72 °C for 5 minutes. Each PCR run included a positive control (diluted genomic DNA), a negative extraction control, and a no-template PCR control. Samples showing no amplification were considered free of genomic DNA and used for cDNA synthesis. PCR products (5 µL) were visualized on a 2% agarose gel after electrophoresis. RNA quantity was measured fluorometrically using the Quant-iT^TM^ RiboGreen^®^ RNA Assay Kit (Thermo Fisher Scientific). To assess RNA integrity and fragment distribution, samples were analyzed on a Fragment Analyzer (Agilent Technologies, Inc.) using the 471 RNA standard kit. Ribosomal Integrity Number (RIN) values were recorded for each sample.

Total RNA (200 ng) was used for first-strand complementary DNA (cDNA) synthesis with the SuperScript™ IV VILO™ Mastermix (Invitrogen™). cDNA libraries were prepared using the NEBNext Ultra II FS DNA Library Prep Kit (New England BioLabs Inc.), following the manufacturer’s protocol. Library purification was performed using AMPure XP magnetic beads (Beckman Coulter Life Sciences, Brea, CA, USA). Quality control and fragment size validation were performed using a Fragment Analyzer (Agilent Technologies, Inc.). Sequencing was conducted on an Illumina NextSeq 550 using the NextSeq High Output Kit v2.5 (2×150 bp paired-end), targeting a sequencing depth of up to 3M paired-end reads per sample.

**Quality Control and Read Processing**

Sequencing read depth was targeted to reach up to 3 million total paired-end reads per sample. Libraries were sequenced using the Illumina NextSeq 550 and the NextSeq High Output Kit v2.5 (Illumina) (2×150 bp, paired-end). Quality control, adapter trimming, and filtering of raw reads were performed with *fastp v0.23.4* [5], resulting in an average of 11 million reads per sample.

**Read Mapping and Transcript Quantification**

A total of 60 samples (2 strains × 3 conditions × 2 time points × 5 replicates) were initially included. Two samples from *B. licheniformis* under BC^plus^ were excluded due to low sequencing quality, resulting in n = 3 for this group. The final dataset comprised 58 samples, with a median of 1.91 × 10^5^ retained reads and 3,655 genes, were included in the final dataset.

Indexes for *Bacillus velezensis* and *Bacillus licheniformis* were generated using *kallisto v0.51.0* [6] with 31 bp k-mers. Reads were pseudo-aligned to the respective indexes with an average alignment rate of 96.6%, resulting in a median of 5 million paired-end reads pseudo-aligned per sample.

**Genome Annotation and Homology Analysis**

The genomes of *Bacillus velezensis* and *Bacillus licheniformis* were annotated using:

- *Bakta v1.8.1* [7]
- *kofamKOALA v2023-06-07* [8] against the KEGG database v106.0 [9]
- *PLaBAse v1.02* [10]

Homologous sequences between genomes were identified using reciprocal best-hits via *mmseqs2 easy-rbh v13.45111* [11].

**Filtering and Normalization**

Reads assigned to rRNA, tRNA, initiation, and elongation factors were removed, alongside genes and samples with low coverage. A total of 55 samples (n = 3–5 per group) with a median of 1.91 × 10⁵ reads and 3,655 retained genes were used for downstream analyses. Counts were transformed into centered log-ratios prior to statistical analysis.

**Statistical and Multivariate Analysis**

Singular value decomposition was performed on the log-ratio matrix to visualize the samples in a low-dimensional ordination. To assess the contribution of experimental variables to sample clustering, Permutational Multivariate Analysis of Variance (PERMANOVA) was conducted using the *vegan v2.6-4* [12] package in R. Generalized linear model was applied to each gene. Post-hoc analyses of the fitted models were performed with the *marginaleffects v0.13.0* [13] package to estimate and compare average marginal effects of the predictors. Pairwise contrasts between all treatment combinations were computed based on gene-wise generalized linear models. Differentially expressed genes were defined as those showing a significant log-ratio difference (FDR adjusted *p* < 0.05) in at least one contrast. All *p* values were adjusted for multiple testing using the Benjamini–Hochberg procedure.

Principal component analysis (PCA) and volcano plots were generated in R v 4.3.1 [2] using either built-in functions or the ggplot2 package. Detailed scripts for all analyses are available at https://github.com/rsiani/thaqi_2025.

## **Metabolite Analysis**

For quantification of metabolites in the supernatants of the three tested treatments, a liquid chromatography coupled to mass spectrometry was used. Supernatants were collected from the same cultures used for RNA-Seq analysis at the first time point (after 6 hours of cultivation). To obtain the supernatant, cultures were centrifuged at 3,750 rpm for 5 minutes, followed by sterile filtration using a 0.22 µm filter. The filtered supernatants were immediately stored at -80°C until further analysis. Proteins from supernatant were precipitated by adding 400 µL acetonitrile to 100 µL of supernatant. After incubation and centrifugation, the cleared sample was transferred to an autosampler vial. Metabolite profiling was performed using a Sciex ExionLC AD coupled to a Sciex ZenoTOF 7600 under the control of Sciex OS 3.0 (Sciex, Darmstadt, Germany). Separation was achieved on an Agilent InfinityLab Poroshell 120 HILIC-Z column (2.1 mm x 150 mm, 2.7 µm particle size, PEEK-lined) (Agilent Technologies, Waldbronn, Germany). Different eluents and gradients were applied for positive and negative ionization modes. In positive ionization mode, eluent A consisted of 100% H_2_O + 10 mM ammonium formate / 0.1% formic acid, and eluent B consisted of 10% H_2_O / 90% ACN + 10 mM ammonium formate / 0.1% formic acid. In negative ionization mode, eluent A consisted of 100% H_2_O + 10 mM ammonium acetate / 2.5 µM medronic acid, pH = 9, and eluent A consisted of 15% H_2_O / 85% ACN + 10 mm ammonium acetate / 2.5 µM medronic acid, pH = 9. The column temperature was set to 25°C and 50°C for positive and negative ionization modes, respectively, and the flow rate was 0.25 mL/min in both cases. Autosampler temperature was set to 5°C and 5 µl were injected for analysis. In MS1, ions in the m/z range 70 to 1500 were accumulated for 0.1s, and information-dependent acquisition (IDA) of MS2 was used with a maximum number of 6 candidate ions and a collision energy of 35 eV with a spread of 15 eV. Accumulation time for MS2 was set to 0.025 seconds, yielding a total cycle time of 0.299 seconds. ZenoTrapping was enabled with a value of 80000. QC samples were used for conditioning the column and injected every ten samples. Automatic calibration of the mass spectrometer in MS^1^ and MS^2^ mode was performed every five injections using the ESI positive Calibration Solution for the Sciex X500 system or the ESI negative Calibration Solution for the Sciex X500 system (Sciex, Darmstadt, Germany). Peak picking was performed using SLAW [29]. Metabolite annotation has been performed by matching against in-house and external MS2 databases using packages from the RforMassSpectrometry infrastructure [30] and Sirius and CSI:FingerID [31, 32].

The metabolomics data were analyzed using R version 4.3.1. Raw data from both positive and negative ionization modes were processed, and statistical analysis was performed using Welch’s t-test to identify significant differences in metabolite abundances between BC^plus^, P limitation, and Full P treatments. For each treatment, five biological replicates were analyzed. Metabolites were considered significantly different if their *p* values were below 0.05 in at least one comparison (BC^plus^ vs. Full or BC^plus^ vs. P limitation). Additionally, only metabolites that showed at least a twofold higher abundance compared to their respective controls were included in the final dataset, ensuring that only strongly differentiated metabolites were considered.

**Table S2** General information and quality of sequenced genomes isolated from bacterial strains

|  | *B. licheniformis* | *B. velezensis* | *P. psychordurans* |
| --- | --- | --- | --- |
| No. of Circularized contigs | 1 | 1 | 2 |
| Chromosome [bp] | 3,674,299 | 3,918,596 | 5,285,600 |
| Plasmid [bp] | - | - | 213,359 |
| GC content [%] | 46.20 | 46.4 | 35.54 |
| Completeness [%] | 98.96 | Not reported | 99.34 |
| Contamination [%] | 0 | Not reported | 1.06 |
| No. of Coding sequences | 3,962 | 3,799 | 5,757 |
| No. of rRNA | 21 | 29 | 37 |
| No. of tRNA | 59 | 89 | 111 |

**Table S3** Identification of bacterial isolates based on whole-genome sequences. Digital DNA–DNA hybridization (dDDH) values and their confidence intervals were calculated based on the total identities within high-scoring segment pairs (HSPs), normalized by the overall HSP length (n=1). Asterisks indicate pairs of strains that were later recognized as identical, although they were originally described as separate strains in previous publications.

| Isolates | Closest type strain | dDDH values | Confidence intervals | Diff. G+C  Percent |
| --- | --- | --- | --- | --- |
| *B. licheniformis* | *Bacillus licheniformis* DSM 13* | 97.4 | 96.3 - 98.1 | 0.02 |
| *P. psychrodurans* | *Psychrobacillus psychrodurans* DSM 11713 | 36.3 | 33.8 - 38.8 | 0.41 |
| *P. psychrodurans* | *Paenisporosarcina quisquiliarum* SK 55 | 36.2 | 33.7 - 38.7 | 0.45 |

**Table S4** Gene set used for manual screening and Venn diagram creation after PLaBAse annotation.

| **Category** | **Gene** | **Function** |
| --- | --- | --- |
| P solubilzation | *gltA, citA, citC, citZ, acnA, acnB, sucA, sucB, sucD, fumABC, mdh, mqo* | Central carbon metabolism – production of organic acids |
|  | *aceF, pdhC, lpd* | Pyruvate dehydrogenase complex – links glycolysis and Tricarboxylic Acid Cycle (TCA) cycle via acetyl-CoA |
|  | *gcd, pqqL, pqqD, ghrB* | Oxidative glucose metabolism – produces gluconic acid for P solubilization |
|  | *citT, citM, citS, oxlT, lutP, lctP, citN, ydiI, menI* | Transport of citrate, oxalate, lactate and other organic acids |
|  | *ackA, pta, acs, poxB, buk, acyP, aceA, aceB, prpE* | Short-Chain Fatty Acids and acetate production, contributes to organic acid pool |
|  | *ldhA, dld, ldf, ldF, ldG, ldR* | Lactate production and regulation |
|  | *frdABCD, fdhD, fdgO, gabD* | Involved in organic acid production and energy metabolism through the conversion of key intermediates |
|  | *maeA, sfcA, ywkA, pyc, pckA* | Organic acid cycling and link to Phosphoenolpyruvate, pyruvate, and malate metabolism |
|  | *ahrA, ahrB, ldR* | Transcriptional regulators of acid production |
| Phytohormone | *ipdC* | Indolepyruvate decarboxylase – key gene in Indole-3-acetic acid (IAA) biosynthesis via indolepyruvate |
|  | *iaaM, iaaH* | Tryptophan monooxygenase / Indole-3-acetamide hydrolase (IAM pathway) |
|  | *amiE* | Amidase – hydrolyzes IAA precursors |
|  | *trpA, trpB, trpC, trpD, trpE, trpF* | Tryptophan biosynthesis genes – precursors for IAA |
|  | *tnaA* | Tryptophanase – produces indole (IAA precursor) |
|  | *aldH* | Aldehyde dehydrogenase – converts IAA-aldehyde to IAA |
|  | *betB* | Possibly linked to IAA metabolism (from PGPT annotation) |
|  | *cps, ks, kaurene synthase, ispa, idi* | Gibberellin precursor synthesis – terpenoid backbone and isoprenoid biosynthesis |
|  | *ipt, miaA* | Isopentenyltransferases – cytokinin biosynthesis and activation |
|  | *log, yvdD* | Cytokinin-activating enzyme and associated factor |
|  | *acdS* | ACC deaminase – reduces plant ACC, suppresses ethylene (stress avoidance) |
|  | *pchA, pchB* | Salicylic acid biosynthesis in *Pseudomonas* |
|  | *speA, speB, speC, speD, speE* | Putrescine, spermidine biosynthesis – involved in growth, stress, possibly signaling |
| S metabolism | *cysA, cysW, cysU, cysT* | ABC transporter complex for sulfate import |
|  | *cysD, cysN, sat* | Sulfate activation to APS (adenylylsulfate) |
|  | *cysC* | Converts APS to PAPS (PAPS kinase) |
|  | *cysH* | Reduces PAPS to sulfite |
|  | *cysI, cysJ* | Sulfite reductase complex – converts sulfite to sulfide |
|  | *cysK, cysM* | Incorporate sulfide into cysteine |
|  | *sqr* | Oxidizes H₂S to elemental sulfur |
|  | *fccA, fccB* | Aerobic oxidation of H₂S |
|  | *soxA, soxB, soxC, soxD, soxX, soxY, soxZ* | Sox multienzyme system – complete oxidation of sulfur compounds |
|  | *sdo* | Converts elemental S to sulfite |
|  | *tetH* | Tetrathionate hydrolase – breaks down tetrathionate |
|  | *dsrA, dsrB, dsrC* | Converts sulfite to sulfide |
|  | *aprA, aprB* | Adenosine 5'-phosphosulfate (APS) reductase – reduces APS to sulfite (dissimilatory) |
|  | *sufS, sufE, sufB, sufC, sufD* | Assembly under oxidative stress or S limitation |
|  | *iscS* | S transferase – supplies S to cofactors and clusters |
|  | *thiS, thiF* | S carriers – thiamine synthesis |
|  | *tusA, tusB, tusC, tusD, tusE* | S transfer for tRNA thiolation |
|  | *yrkH, yrkI, yrkJ* | Regulatory proteins in sulfur oxidation |
|  | *spxH* | Redox-responsive transcription factor for sulfur genes |
|  | *cymR* | Global repressor of S metabolism |

**Table S5** Screened genes related to bacterial P turnover.

| **Category** | **Genes** | **Function** |
| --- | --- | --- |
| Low-affinity phosphate transporter | *pitA, pitB* | Constitutive Pi uptake via low-affinity transport |
| High-affinity phosphate transporter (Pst system) | *pstS, pstC, pstA, pstB* | ABC-type inorganic P transporter system, active under P limitation |
| Alternate high-affinity inorganic phosphate transporter | *appA, appB, appC, appD, appF* | Alternative ABC transporter for inorganic P |
| Glycerol-3-phosphate transporter | *ugpA, ugpB, ugpC, ugpE, ugpQ* | Uptake of glycerol-3-phosphate, a source of organic phosphate |
| Phosphonate transport and metabolism | *phnC, phnD, phnE, phnF, phnG, phnH, phnI, phnJ, phnK, phnL, phnM, phnN, phnO, phnP, phnW, phnX, phnA* | Transport and cleavage of phosphonates (C–P bond) |
| Polyphosphate metabolism | *ppk1, ppk2, ppaC, ppaX* | Polyphosphate synthesis (PPK), degradation (PPA), P storage and release |
| Alkaline phosphatases | *phoA, phoD, phoX* | Mineralization of organic phosphorus under alkaline pH |
| Acid phosphatases / Phytases | *phy, appA, agp* | Release of inorganic P from phytate and other organic compounds at low pH |
| Pho regulon – regulatory system | *phoB, phoR, phoP, phoQ, phoU, phoH* | Two-component systems regulating P starvation response |

**Table S6** Gene features involved in P turnover across the four strains. Numbers in parentheses indicate the number of gene copies identified per strain.

|  |  | *B. licheniformis* | *B. velezensis* | *P. pychrodurans* |
| --- | --- | --- | --- | --- |
| P transport | Inorganic P | *pitA* (3)*, pstA* (1)*, pstB* (2)*, pstC* (1)*, pstS* (1) | *appF* (1), *pit* (1)*, pstA* (1)*, pstB* (2)*, pstC* (1)*, pstS* (1) | *pstS* (3), *pstB* (2), *pstC* (2), *pstA* (2) |
|  | Organic P | *ugpA* (5)*, ugpB* (5)*, ugpE* (4)*, ugpQ* (4) | *ugpC* (1)*, ugpQ* (3) | *ugpA* (1), *ugpC* (1), *ugpE* (1), *phnE* (1), *phnG* (1), *phnH* (1), *phnI* (1), *phnJ* (1), |
| Inorganic P |  | *phoE* (2)*, ppaC* (1), *ppaX* (1)*, appA* (1) | *ppaC* (1), *ppaX* (1) | *ppaX* (1) |
| Organic P |  | *phoA* (1), *phoD* (1)*, phy* (1), *phn* | *phoD* (1), *phnF* (1)*, phyC* (1), | *phnA (1), phnB phnB* (2) |
| P regulation |  | *phoR* (2), *phoB* (2), *phoP* (1), *phoH* (2) | *phoR* (1), *phoB* (1), *phoP* (1), *phoH* (1) | *phoR* (1), *phoB* (1), *phoP* (1), *phoU* (3), *phoH* (1) |


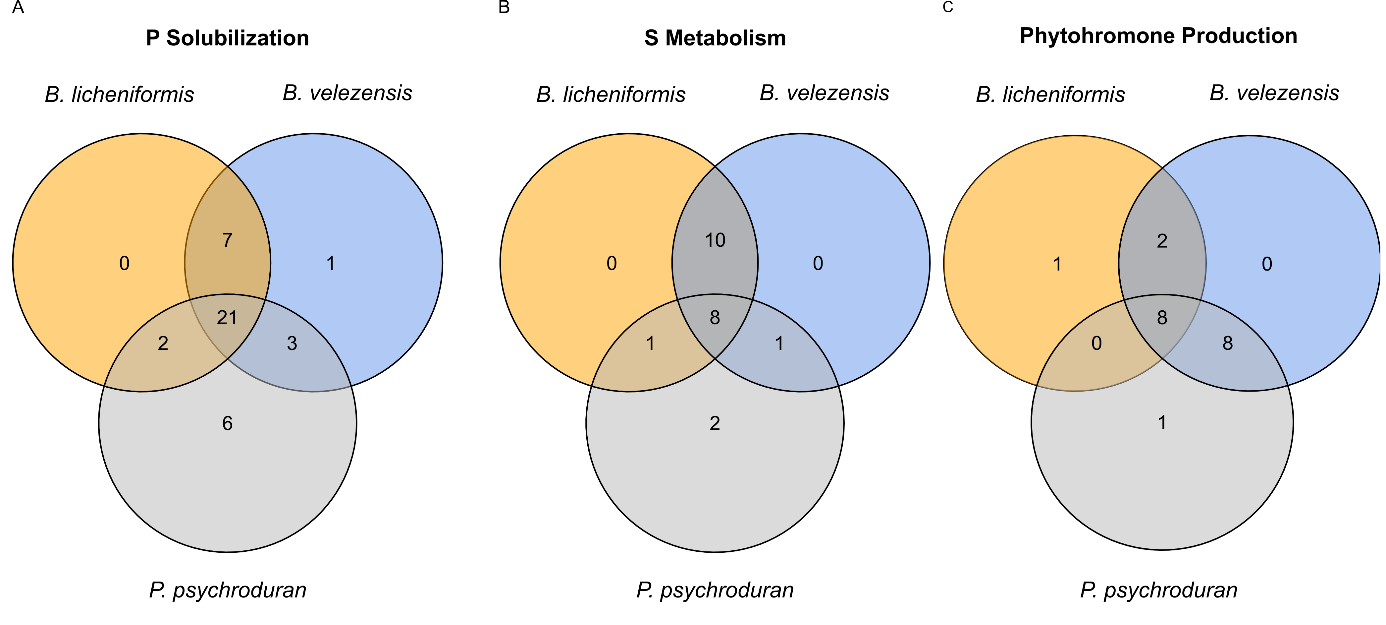


**Figure S1** Distribution of functional genes related to (A) P solubilization, (B) S metabolism, and (C) phytohormone production across three bacterial strains (Bacillus licheniformis, Bacillus velezensis, and Pseudomonas psychrodurans). Numbers indicate the common and unique genes annotated for each functional category based on PLaBAse predictions and manual filtering.


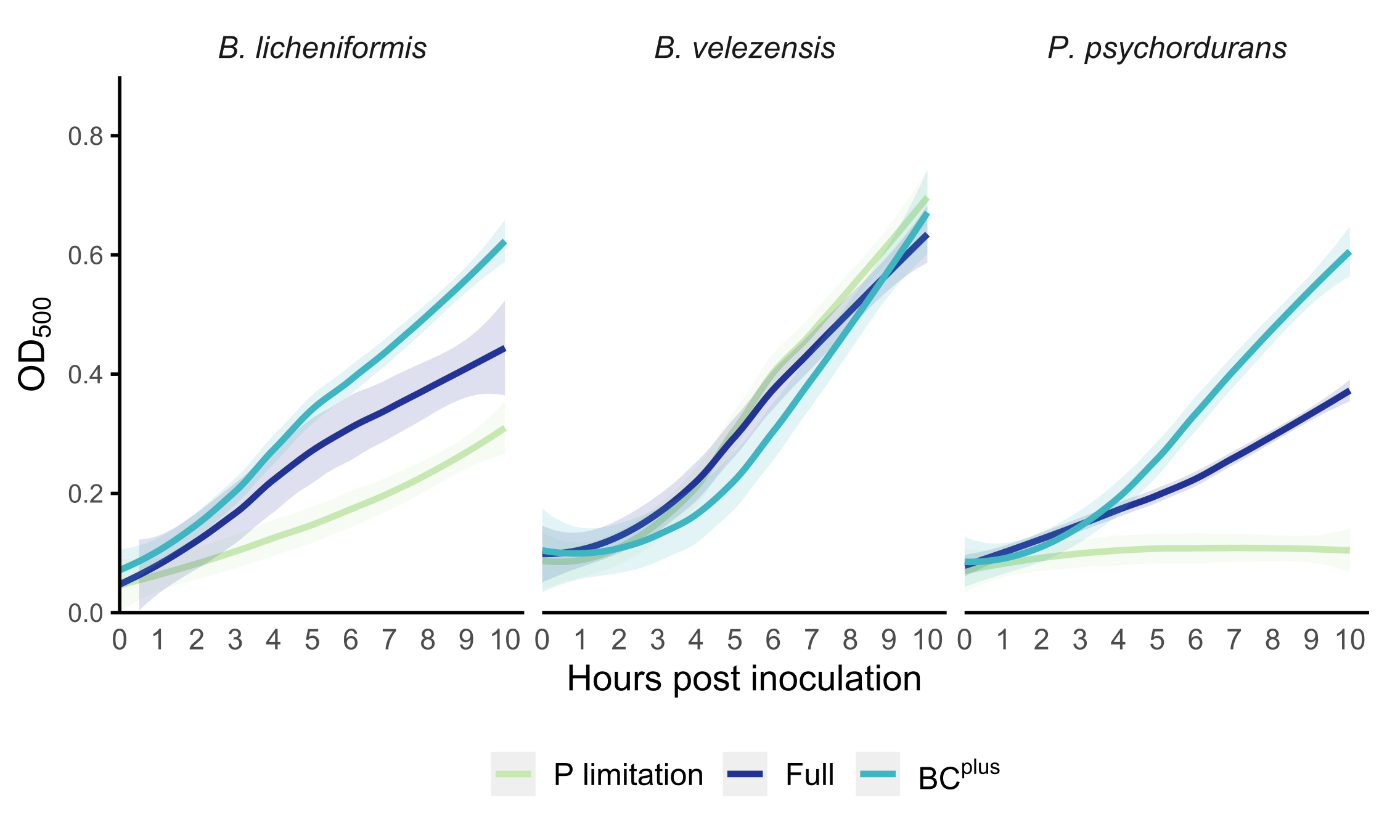


**Figure S2** Growth curves of B. licheniformis, B. mycoides, B. velezensis, and P. psychrodurans cultivated in BMM under three different P conditions: P limitation (green), full (blue), and BC^plus^ (cyan). Growth was measured as optical density at 500 nm (OD_500_) over 10 hours period for two independent experimental days (n = 3 per day). Solid lines represent the LOESS smoothed means; shaded areas indicate the standard error of the mean (SEM) from three biological replicates.

**Table S7** Summary of PERMANOVA results assessing the effect of P source, Timepoint and Strain on transcriptome profiles. The analysis was performed across all samples from both strains, and separately for B. licheniformis and B. velezensis.

| **Dataset** | **Term** | **df** | **SumOfSqs** | **R^2^** | **Statistic** | ***p* value** |
| --- | --- | --- | --- | --- | --- | --- |
| Both strains | P source | 2 | 15295.61 | 0.0188 | 2.8121 | 0.039 |
|  | Strain | 1 | 56258.4 | 0.6929 | 27.53202 | 0.001 |
|  | Time point | 3 | 35140.94 | 0.0435 | 12.96315 | 0.002 |
|  | P source:Strain | 2 | 12954.24 | 0.016 | 2.389348 | 0.004 |
|  | P source:Time point | 2 | 1951 | 0.0024 | 0.3517 | 0.936 |
|  | Strain:Timepoint | 3 | 3076.07 | 0.0038 | 1.0228 | 0.009 |
|  | P source:Strain:Time point | 2 | 2023.57 | 0.0025 | 0.3728 | 0.701 |
|  | Residual | 43 | 116565.8 | 0.1436 |  |  |
|  | Total | 54 | 811553.6 | 1 |  |  |
| *B. licheniformis* | P source | 2 | 7776.85 | 0.116366 | 2.950338 | 0.007 |
|  | Time point | 1 | 25903.8 | 0.387602 | 19.65448 | 0.001 |
|  | P source:Time point | 2 | 6791.116 | 0.101616 | 2.576376 | 0.012 |
|  | Residual | 20 | 26359.18 | 0.394416 |  |  |
|  | Total | 25 | 66830.95 | 1 |  |  |
| *B. velezensis* | P source | 2 | 10505.61 | 0.155877 | 3.0097 | 0.006 |
|  | Timepoint | 1 | 7348.15 | 0.109082 | 4.210272 | 0.007 |
|  | P source:Time point | 2 | 9401.231 | 0.139491 | 2.693313 | 0.006 |
|  | Residual | 23 | 40141.7 | 0.595603 |  |  |
|  | Total | 28 | 67396.68 | 1 |  |  |

**Table S8** Analysis of Variance (ANOVA) results for P mobilization from BC^plus^. The table presents the results of the generalized least squares (GLS) model evaluating the effects of strain, time, and their interaction on P mobilization from BC^plus^. Degrees of freedom (numDF), F values, and p values are reported. Significant effects (p < 0.05) are highlighted.

| **Factor** | **numDF** | **F value** | ***p* value** |
| --- | --- | --- | --- |
| Intercept | 1 | 4090.107 | <.0001 |
| Strain | 2 | 454.009 | <.0001 |
| Time | 5 | 485.409 | <.0001 |
| bioReplicate | 1 | 9.092 | 0.003 |
| Strain:Time | 10 | 95.053 | <.0001 |

**Table S9** Post-hoc Tukey-adjusted pairwise comparisons of P mobilization from BC^plus^ across strains and time points. The table presents pairwise comparisons between strains at each time point using Tukey-adjusted estimated marginal means (emmeans). Reported values include the estimated difference (Estimate), standard error (SE), degrees of freedom (DF), t ratio, and p value.

| **Time** | **Contrast** | **Estimate** | **SE** | **DF** | **t ratio** | ***p* value** |
| --- | --- | --- | --- | --- | --- | --- |
| 0 | Control - *B.velezensis* | -0.15994 | 0.0896 | 43.2 | -1.785 | 0.1866 |
|  | Control - *B.licheniformis* | -0.067 | 0.0896 | 43.2 | -0.748 | 0.7365 |
|  | *B.velezensis*- B.licheniformis | 0.09294 | 0.0896 | 43.2 | 1.037 | 0.5577 |
| 3 | Control - *B.velezensis* | -0.25621 | 0.0896 | 73 | -2.86 | 0.0151 |
|  | Control - *B.licheniformis* | -0.21644 | 0.0896 | 73 | -2.416 | 0.0473 |
|  | *B.velezensis*- B.licheniformis | 0.03977 | 0.0896 | 73 | 0.444 | 0.8973 |
| 6 | Control - *B.velezensis* | -0.25163 | 0.0896 | 73.1 | -2.808 | 0.0173 |
|  | Control - *B.licheniformis* | -0.24464 | 0.0896 | 73.1 | -2.73 | 0.0214 |
|  | *B.velezensis*- B.licheniformis | 0.00699 | 0.0896 | 73.1 | 0.078 | 0.9967 |
| 24 | Control - *B.velezensis* | -1.35912 | 0.0896 | 73.1 | -15.169 | <.0001 |
|  | Control - *B.licheniformis* | -0.46156 | 0.0896 | 73.1 | -5.151 | <.0001 |
|  | *B.velezensis*- *B.licheniformis* | 0.89757 | 0.0896 | 73.1 | 10.018 | <.0001 |
| 48 | Control - *B.velezensis* | -2.38612 | 0.0896 | 73 | -26.632 | <.0001 |
|  | Control - *B.licheniformis* | -2.47289 | 0.0896 | 73 | -27.6 | <.0001 |
|  | *B.velezensis*- *B.licheniformis* | -0.08677 | 0.0896 | 73 | -0.968 | 0.599 |
| 72 | Control - *B.velezensis* | -2.41986 | 0.0896 | 43.2 | -27.008 | <.0001 |
|  | Control - *B.licheniformis* | -2.36201 | 0.0896 | 43.2 | -26.363 | <.0001 |
|  | *B.velezensis*- *B.licheniformis* | 0.05785 | 0.0896 | 43.2 | 0.646 | 0.7958 |


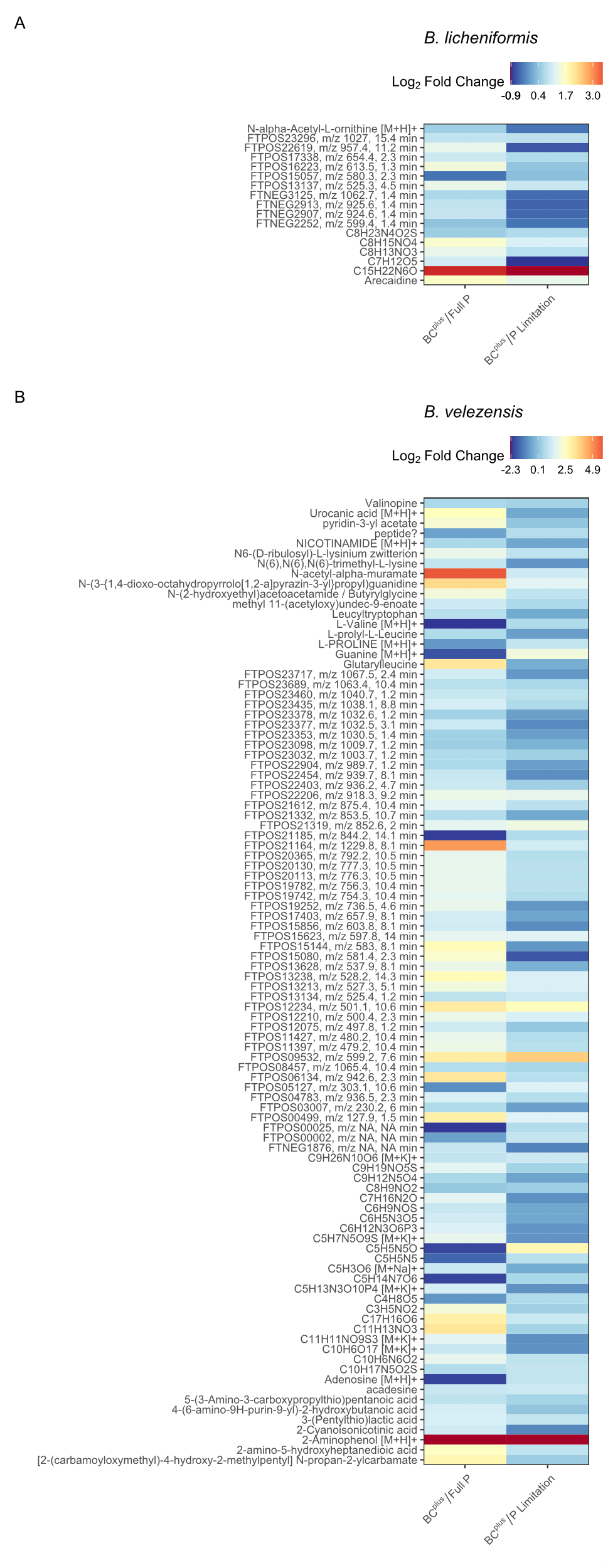


**Figure S4** Significant secreted metabolites from B. licheniformis and B. velezensis. Heatmaps display the log_2_ fold change of significant metabolites detected in BC^plus^ medium inoculated with B. licheniformis (A) and B. velezensis (B) compared to the controls (P limitation and Full P) after 6 hours. The color scale represents fold-change values, with blue indicating downregulation and red indicating upregulation. For unknown metabolites, the respective m/z and retention time (RT) are indicated. All metabolite annotations are putative.

**References:**

1. Stülke J, Hanschke R, Hecker M. Temporal activation of beta-glucanase synthesis in *Bacillus subtilis* is mediated by the GTP pool. J Gen Microbiol 1993;139:2041–5. https://doi.org/10.1099/00221287-139-9-2041

2. R Core Team. R: A language and environment for statistical computing. Vienna: R Foundation for Statistical Computing; 2023

3. Zimmer D, Kruse J, Siebers N, et al. Bone char vs. S-enriched bone char: Multi-method characterization of bone chars and their transformation in soil. Sci Total Environ 2018;643:145–56. https://doi.org/10.1016/j.scitotenv.2018.06.076

4. Chen XH, Koumoutsi A, Scholz R, et al. Comparative analysis of the complete genome sequence of the plant growth–promoting bacterium *Bacillus amyloliquefaciens* FZB42. Nat Biotechnol 2007;25:1007–14. https://doi.org/10.1038/nbt1325.

5. Chen S, Zhou Y, Chen Y, et al. fastp: an ultra-fast all-in-one FASTQ preprocessor. Bioinformatics 2018;34:i884–90. https://doi.org/10.1093/bioinformatics/bty560.

6. Bray NL, Pimentel H, Melsted P, et al. Near-optimal probabilistic RNA-seq quantification. Nat Biotechnol 2016;34:525–7. https://doi.org/10.1038/nbt.3519

7. Schwengers O, Jelonek L, Dieckmann MA, et al. Bakta: rapid and standardized annotation of bacterial genomes via alignment-free sequence identification. Microb Genom 2021;7. https://doi.org/10.1099/mgen.0.000685

8. Aramaki T, Blanc-Mathieu R, Endo H, et al. KofamKOALA: KEGG Ortholog assignment based on profile HMM and adaptive score threshold. Bioinformatics 2020;36:2251–2. https://doi.org/10.1093/bioinformatics/btz8599. Kanehisa M, Furumichi M, Tanabe M, et al. KEGG: new perspectives on genomes, pathways, diseases and drugs. Nucleic Acids Res 2017;45:D353–61. https://doi.org/10.1093/nar/gkw1092

10. Patz S, Gautam A, Becker M, et al. PLaBAse: A comprehensive web resource for analyzing the plant growth-promoting potential of plant-associated bacteria. bioRxiv 2021. https://doi.org/10.1101/2021.12.13.47247111. Steinegger M, Söding J. MMseqs2 enables sensitive protein sequence searching for the analysis of massive data sets. Nat Biotechnol 2017;35:1026–8. https://doi.org/10.1038/nbt.3988

12. Oksanen J, Blanchet FG, Kindt R, et al. vegan: Community Ecology Package. R package version 2.6-4. 2022.

13. Arel-Bundock V. marginaleffects: Predictions, comparisons, slopes, marginal means, and hypothesis tests. R package version 0.17.0. 2023.
